# AMPK Suppression Due to Obesity Drives Oocyte mtDNA Heteroplasmy via ATF5‐POLG Axis

**DOI:** 10.1002/advs.202307480

**Published:** 2024-03-18

**Authors:** Yanting Chen, Guiling Ma, Yang Gai, Qiyuan Yang, Xiangdong Liu, Jeanene M. de Avila, Shengyong Mao, Mei‐Jun Zhu, Min Du

**Affiliations:** ^1^ National Center for Internatinal Research on Animal Gut Nutrition Jingsu Key Laboratory of Gastrointestinal Nutrition and Animal Health College of Animal Science and Technology Nanjing Agricultural University Nanjing 210095 China; ^2^ Nutrigenomics and Growth Biology Laboratory Department of Animal Sciences Washington State University Pullman WA 99164 USA; ^3^ Department of Molecular Cell and Cancer Biology University of Massachusetts Chan Medical School Worcester MA 01655 USA; ^4^ Department of Cancer biology Dana‐Farber Cancer Institute Harvard Medical School Boston MA 02215 USA; ^5^ School of Food Sciences Washington State University Pullman WA 99164 USA

**Keywords:** AMPK, female obesity, mature oocyte, mtDNA heteroplasmy

## Abstract

Due to the exclusive maternal transmission, oocyte mitochondrial dysfunction reduces fertility rates, affects embryonic development, and programs offspring to metabolic diseases. However, mitochondrial DNA (mtDNA) are vulnerable to mutations during oocyte maturation, leading to mitochondrial nucleotide variations (mtSNVs) within a single oocyte, referring to mtDNA heteroplasmy. Obesity (OB) accounts for more than 40% of women at the reproductive age in the USA, but little is known about impacts of OB on mtSNVs in mature oocytes. It is found that OB reduces mtDNA content and increases mtSNVs in mature oocytes, which impairs mitochondrial energetic functions and oocyte quality. In mature oocytes, OB suppresses AMPK activity, aligned with an increased binding affinity of the ATF5‐POLG protein complex to mutated mtDNA D‐loop and protein‐coding regions. Similarly, AMPK knockout increases the binding affinity of ATF5‐POLG proteins to mutated mtDNA, leading to the replication of heteroplasmic mtDNA and impairing oocyte quality. Consistently, AMPK activation blocks the detrimental impacts of OB by preventing ATF5‐POLG protein recruitment, improving oocyte maturation and mitochondrial energetics. Overall, the data uncover key features of AMPK activation in suppressing mtSNVs, and improving mitochondrial biogenesis and oocyte maturation in obese females.

## Graphical abstract

mtDNA heteroplasmy with obese fertility Obesity induces AMPK inactivation, lower mtDNA quantity and quality in mature oocytes, attributing to impaired mitochondrial energetics and female fertility. AMPK inactivation increases binding affinity of ATF5‐POLG protein complex to D‐loop and protein‐coding regions of mutated mtDNA, leading to replication of heteroplasmic mtDNA, impaired mitochondrial biogenesis and oocyte quality. AMPK activation blocks the detrimental impacts of OB by preventing ATF5‐POLG protein recruitment, benefiting oocyte maturation and fertility.

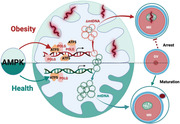

## 1 Introduction

In recent decades, obesity (OB) has become a pandemic.^[^ ^1^ ^]^ Particularly, over two‐thirds of females at their reproductive age are either overweight or OB, and the number shows no signs of decline.^[^ ^2^ ^]^ Beyond its impacts on metabolic health, female OB at reproductive age also reduces oocyte quality,^[^ ^3^ ^]^ contributing to decreased fertility rates worldwide, even in conjunction with assisted reproductive techniques like in vitro fertilization.^[^ ^4^ ^]^ Besides, the suboptimal oocyte maturation due to OB may alter the developmental trajectories of fetal tissues and organs, predisposing offspring to various metabolic and neurological disorders.^[^ ^5^ ^]^ Thus, unraveling the underlying mechanisms linking OB to impaired oocyte maturation is critical for enhancing both maternal fertility and the health of future generations born to obese mothers.

Following puberty, oocytes mature within follicles through undergoing two meiotic divisions accompanied by marked size expansion, necessitating substantial structural and biochemical transformations that demand energy.^[^ ^6^ ^]^ Such energy is primarily provided by mitochondrial DNA (mtDNA), which encode 13 vital subunits of respiration chain complexes.^[^ ^7^ ^]^ As oocyte maturation progresses, there is a profound surge in mtDNA content, escalating from a few hundreds to over 1.5×10^6^ copies per oocyte.^[^ ^8^ ^]^ Although mitochondria own the machinery required for DNA damage repairing, including proofreading activity of mtDNA polyermase γ (POLG), the DNA repair in mitochondria is not robust as in the nucleus.^[^ ^9^ ^]^ Meanwhile, mtDNA are also lack of histone protection against free radical damage, and spontaneous base hydrolysis and/or POLG causes mtDNA replication errors;^[^ ^10^ ^]^ as a result, the rapid mtDNA replications during oocyte maturation may dramatically increase mitochondrial nucleotide variances (mtSNVs) and related mutations, termed mtDNA heteroplasmy.^[^ ^11^ ^]^ Metabolic dysfunctions, such as aging and hypoxia, can accelerate mtDNA heteroplasmy in mature oocytes, which compromise female fertility.^[^ ^12^ ^]^ Considering the exclusive transmission of mtDNA through the maternal germ line, even marginal levels of mtDNA heteroplasmy in mature oocytes augment the risk of mitochondria‐associated diseases in offspring, such as type 2 diabetes and Parkinson disease.^[^ ^13^ ^]^ Despite the prevalence of maternal OB, the impacts of OB on mtDNA heteroplasmy in mature oocytes remain undefined.

Recently, based on studies with *C. elegans*, a mitochondrial unfolded protein responses (UPR^mt^) effector, transcription factor 5 (ATF5/ATFS‐1), was discovered to regulate mutated mtDNA replication by recruiting POLG preferentially to mutated mtDNA.^[^ ^14^ ^]^ Consistently, the activation of ATF5 due to a point mutation increased mtDNA heteroplasmy, contributing to osteoporosis in human patients.^[^ ^15^ ^]^ LonP1 emerges as a dominant protease in mitochondria, which is responsible for clearing unfolded or misfolded mitochondrial proteins.^[^ ^16^ ^]^ LonP1 degrades ATF5, and ATF5 accumulation inside mitochondria increases mutated mtDNA replication.^[^ ^17^ ^]^ However, LonP1 is only active in the presence of adequate ATP level.^[^ ^13^ ^]^ AMP‐activated protein kinase (AMPK) is a master regulator of energy metabolism, and its activation stimulates ATP synthesis.^[^ ^18^ ^]^ On the other hand, AMPK inactivation leads to oocyte aging, germ vesicle breakdown arrest and meiotic maturation blockades.^[^ ^19^ ^]^ We previously found that AMPK activity is robustly inhibited in fetal tissues due to maternal OB, including adipose tissue, skeletal muscle and placenta.^[^ ^20^ ^]^ However, the roles of AMPK in mediating oocyte maturation and mtDNA heteroplasmy of OB females remain unexamined. We hypothesized that OB suppresses AMPK activity, which dysregulates ATP‐dependent LonP1 protease and ATF5‐associated UPR^mt^, resulting in excessive mutated mtDNA replication and disrupting oocyte maturation.

In the present study, we discovered that OB impairs the female fecundity and oocyte maturation, and reduces mtDNA content and increases mtSNVs in mature oocytes. These mutations are concentrated in D‐loop and protein coding genes of mtDNA, together with reduced mtDNA content, potentially explaining the impaired mitochondrial biogenesis and energetics. Meanwhile, OB inactivates AMPK, which disrupts the LonP1‐ATF5 axis and contributes to mtSNVs accretion in mature oocytes. We found that AMPK activation can suppress the dysregulation of LonP1‐ATF5 axis in OB oocytes, consequently limiting mtDNA mutations and restoring mitochondrial energetics and oocyte maturation. Our data identified that AMPK inactivation is a driver of mitochondrial biogenesis and mtDNA heteroplasmy via the LonP1‐ATF5 axis in mature oocytes, providing a therapeutic target to improve oocyte maturation and female fertility, as well as offspring metabolic health, which are impaired due to obesity.

## 2 Results

### 2.1 Obesity Impairs Oocyte Maturation and Mitochondrial Energetics

**Figure 1:**
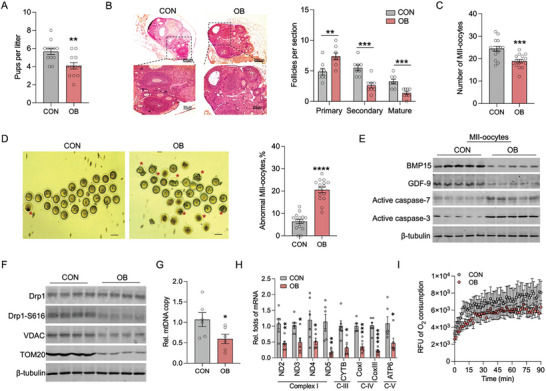
Obesity impairs oocyte maturation and mtDNA activity. A) Number of pups per litter born from female mice fed control (CON) and obesogenic diet (OB) (n = 12). B) H&E staining of ovaries and follicles. Scale bar 200 µm, 50 µm (n = 8). The number of primary, secondary and antral follicles per section of ovary in CON and OB females (n = 8). C, D) The mature oocytes were collected in the fallopian tubes, and the number of MII oocytes and the proportion of abnormal MII oocytes were counted. Stars indicate the abnormal oocytes, and scale bar 50 µm (n = 15). E) Immunoblotting of oocyte maturation indicators in MII oocytes, including BMP15, GDF‐9, active caspase‐7, and active caspase‐3. β‐Tubulin was used as a loading control (n = 5). F) Immunoblotting of mitochondrial fission and biomass indicators in MII oocytes, including Drp1, Drp1‐S616, VDAC and Tom20. β‐Tubulin was used as a loading control (n = 5). G) mtDNA content in mature oocytes (n = 6). H) mRNA expression of mtDNA genes involved in respiration chain complex (n = 6). I) O_2_ consumption was measured in mature oocytes (n = 5; 70 oocytes were pooled per replicate). Data are presented as mean ± s.e.m. *P < 0.05, **P < 0.01, ***P < 0.001, and ****P < 0.0001; unpaired two‐tail Student's t test was used in analyses.

Following an 8‐week high fat diet (HFD) to become obesity (OB) (Figure S1A, Supporting Information), female mice showed reduced fertility, with an average litter size of 3.8 pups per mating compared to 5.7 pups in normal‐weight females (**Figure** **1**A). Aligned with the reduced fertility, ovaries in obese mice exhibited fewer secondary and antral follicles surrounded by multilayered granulosa cells (Figure 1B). After superovulation, fewer mature oocytes were recovered from the oviducts of OB females (Figure 1C). The mature oocytes of OB females also displayed abnormal morphology (Figure 1D), accompanied by decreased BMP‐15 and GDF‐9 proteins and increased Caspase 7 and Caspase 3 apoptotic protein levels (Figures 1E and S1B–E, Supporting Information),^[^ ^21^ ^]^ showing an impaired oocyte maturation in OB females.

Mitochondrial oxidative phosphorylation (OXPHOS) is essential for oocyte maturation^.[^ ^22^ ^]^ Immunostaining with MitoSpy‐Green and TMRE showed that OB not only reduced mitochondrial density, but also substantially suppressed mitochondrial membrane potential, along with the accumulation of abnormal mitochondrial aggregates (Figure S2A–C, Supporting Information).^[^ ^23^ ^]^ Drp1 activity, responsible for mitochondrial fission, was highly inhibited in OB oocytes, consistent with the reduced levels of mitochondrial membrane pore proteins VDAC and TOM20 (Figure 1F; Figure S2D, Supporting Information), showing impaired mitochondrial function. To be an essential energetic component, mtDNA content and transcription of its‐encoded genes were notably inhibited in OB oocytes (Figure 1G,H), which were aligned with less oxygen consumption rates (Figure 1I; Figure S2E, Supporting Information), indicating substantial mitochondrial energetic dysfunction in OB oocytes.

### 2.2 Excessive Heteroplasmy and mtSNVs in Mature Oocytes of OB Females

**Figure 2:**
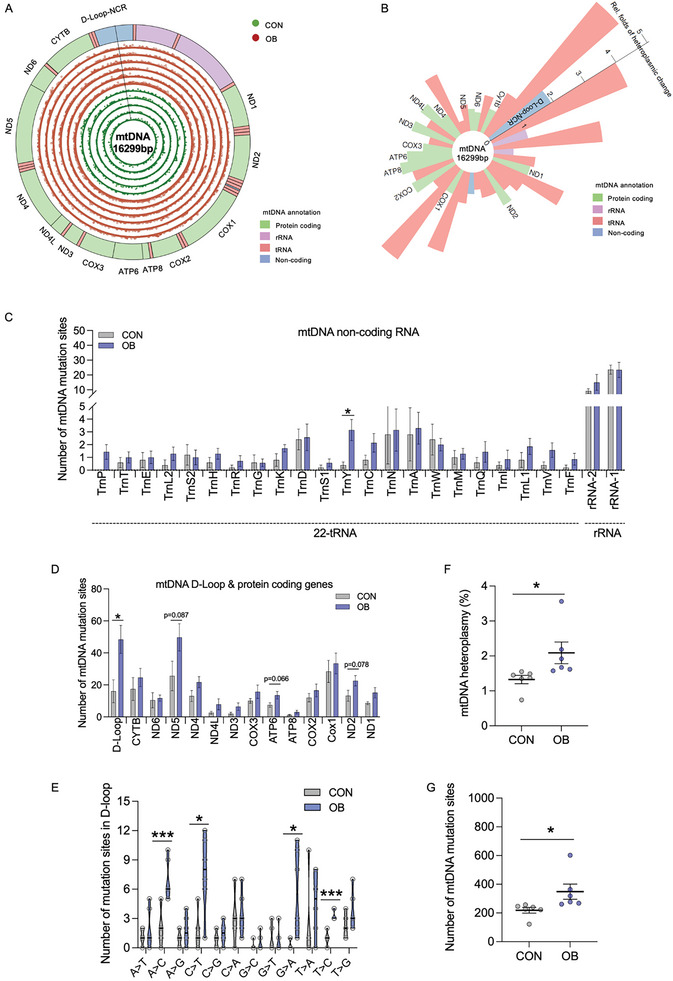
Obesity increases mtDNA heteroplasmy in D‐loop and protein‐coding genes. A) Circular plot of the mitochondrial genome shows annotation on the outer circle. Five‐green inner circles and six‐red outside circles show the genomic locations and the relative frequencies of mtDNA mutations in mature oocytes of control (CON; green color) and obese (OB; red color) females, respectively. Regions corresponding to the different mtDNA genes (green, protein coding genes; pink, rRNA; red, tRNA; blue, non‐coding regions). B) Relative folds of mutation rates in various mtDNA genomic regions. The heteroplasmic change was OB compared with controls. Regions corresponding to the different mtDNA genes (green, protein coding genes; pink, rRNA; red, tRNA; blue, non‐coding regions). C, D) Number of mtDNA mutation sites in mtDNA non‐coding RNAs (C), D‐Loop and protein coding‐genes (D) (n = 5–6). E) Number of mutation sites in D‐loop region (n = 5–6). F, G) The percentage of mtDNA heteroplasmy (F), and number of mutation sites (G) in mature oocytes (n = 5–6). Data are presented as mean ± s.e.m. *P < 0.05, **P < 0.01; unpaired two‐tail Student's t test was used in analyses.

Besides mtDNA content, excessive production of mtDNA heteroplasmy can reduce mtDNA transcription and OXPHOS, impairing mitochondrial energetics and oocyte maturation.^[^ ^11b^ ^]^ Sequencing of mtDNA in mature oocytes from both normal and OB females revealed a dominant presence of mtSNVs in D‐loop, and tended to increase mtSNVs in protein coding genes including *Nd5*, *Atp6* and *Nd2*, while non‐coding RNAs, particularly tRNA, barely showed mutations in mature oocytes (**Figure** **2**A–D). Mutations were existed as single nucleotide variations, and deletions were not observed in both normal and OB oocytes (Figure 2E). Notably, OB females had significantly higher heteroplasmic mtDNA and total mutation sites compared to normal females (Figure 2F,G). Aligned with transcriptional inactivation (Figure 1H), mtSNVs were not only enriched in coding genes, but also localized in the D‐loop and some tRNA regions, including TrnY (Figure 2B–D). After assessing the mutation types in D‐loop, we further noticed both nucleotide transition (C>T, G>A, T>C) and transversion (A>C) mutations were highly existed in OB oocytes (Figure 2E). Besides, the mtDNA heteroplasmy was also closely associated with reduced oxygen consumption rate (Figure S2F, Supporting Information), indicating the impaired mtDNA quantity and quality potentially link to mitochondrial dysfunctions in mature oocytes of OB females.

### 2.3 Dysregulation of ATF5‐POLG‐mtDNA Heteroplasmy Axis in OB Oocytes

**Figure 3:**
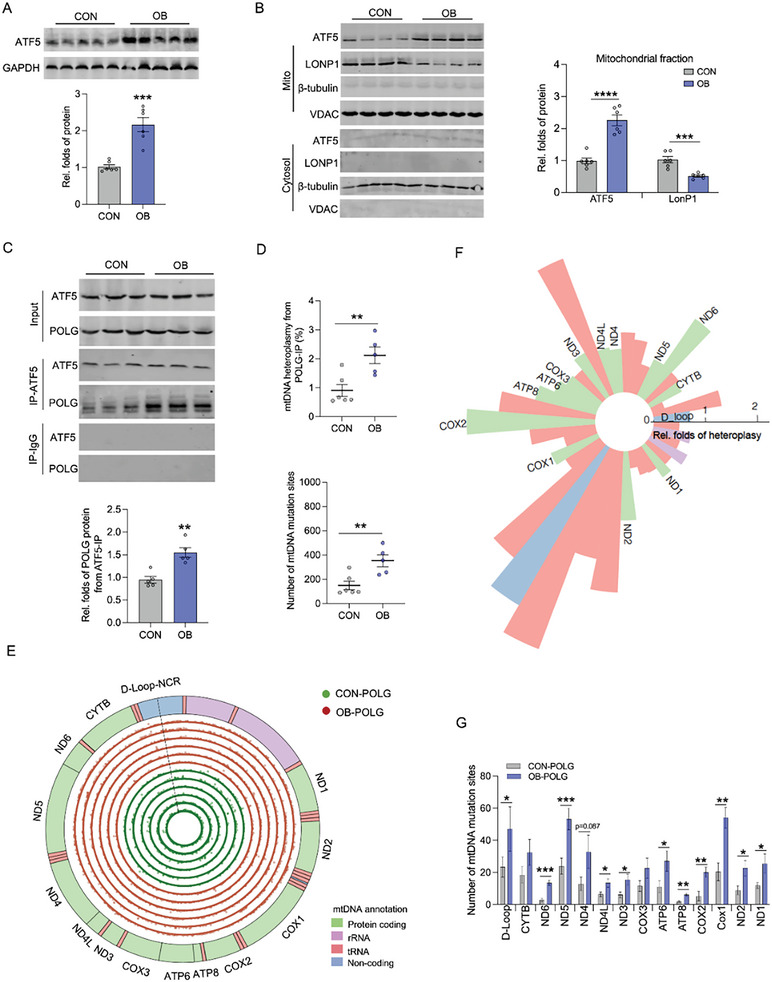
ATF5‐POLG protein axis dysregulates mtDNA heteroplasmy in mature oocytes. A) Immunoblotting of ATF5 in mature oocytes of control (CON) and obese (OB) females. GAPDH was used as a loading control (n = 6). B) Immunoblotting of ATF5 and LonP1 proteins in cytosol and mitochondria of mature oocytes. VDAC was used as mitochondrial loading control, and β‐tubulin was used as a cytosol loading control (n = 6). C) Co‐immunoprecipitation of ATF5 in measuring POLG in mature oocytes. ATF5 and POLG were immunoprecipitated followed with SDS‐PAGE separation and measured by immunoblotting. IgG was used as a negative control in immunoprecipitation (n = 6). D) The percentage of mtDNA heteroplasmy and total mutation sites in POLG‐immunoprecipitants (n = 5–6). E, F) Circular plot of the mitochondrial genome shows the genome annotation on the outer circle. The six green inner circles and six red outside cycles show the genomic locations and the relative frequencies of mtDNA mutations from POLG immunoprecipitants in mature oocytes of CON (green, inner cycle) and OB females (red, outside cycle) (E). Relative fold changes of mtDNA heteroplasmy between CON and OB mature oocytes were mapped (F). The heteroplasmic change was OB compared with controls. Regions corresponding to the different mtDNA genes (green, protein coding genes; pink, rRNA; red, tRNA; blue, non‐coding regions) (n = 5–6). G) Total mutation sites in D‐loop and protein coding genes of mtDNA from POLG immunoprecipitate in mature oocytes (n = 5–6). Data are presented as mean ± s.e.m. *P < 0.05, **P < 0.01, ***P < 0.001, and ****P < 0.0001; unpaired two‐tail Student's t test was used in analyses.

ATF5 has been identified as a key activator of mitochondrial unfolding protein responses (UPR^mt^) in initiating heteroplasmic mtDNA replications by recruiting POLG to mutated mtDNA.^[^ ^14^, ^15^ ^]^ In OB females, ATF5 protein was higher in mature oocytes (**Figure** **3**A) and mainly localized in mitochondria (Figure 3B). Consistent with ATF5 activation, UPR^mt^ indicators, including HSP70, Clpp, Hspd1 and Chop, were increased in OB oocytes (Figure S3A–C, Supporting Information). Using ATF5‐immunoprecipitation assay, OB oocytes had more POLG binding to ATF5 protein, despite similar levels of total POLG protein between normal and OB oocytes (Figure 3C; Figure S3D, Supporting Information). Furthermore, using POLG immunoprecipitation of mtDNA fragments for sequencing, OB oocytes had more mutated mtDNA bound to POLG protein (Figure 3D,F) and the binding preferably located in the mutated D‐loop and protein‐coded genes (Figure 3G). Similarly, ATF5 immunoprecipitation showed higher content of mutated mtDNA bound to ATF5 protein in OB oocytes, and the mutation was preferentially located in D‐loop and protein‐coding regions (Figure S3E–G, Supporting Information).

**Figure 4:**
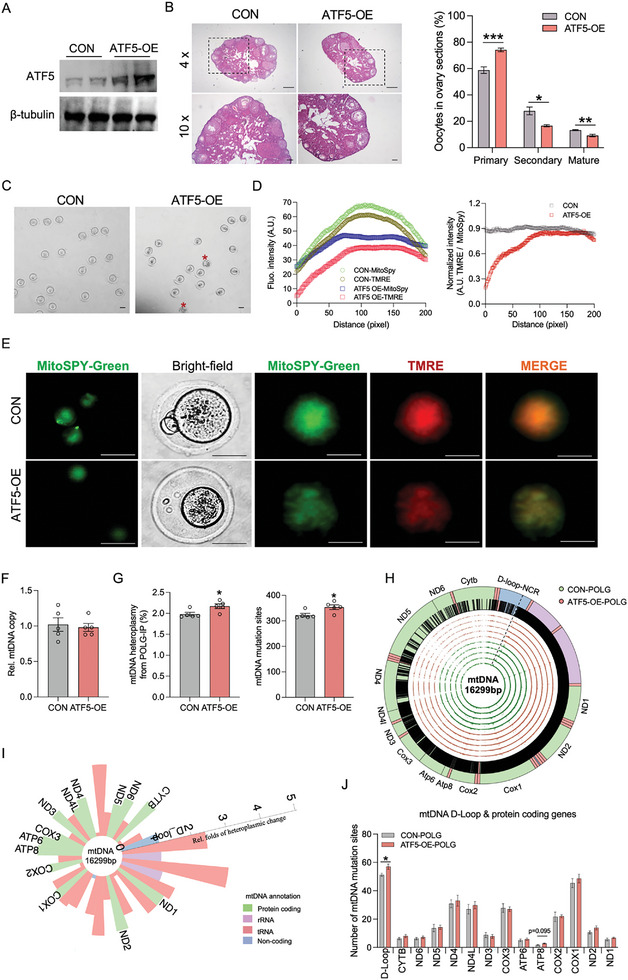
ATF5 activation impairs oocyte maturation and increases mtDNA heteroplasmy. A) 3‐month old mice were administrated AAV9‐MCS‐CMV (CON) or AAV9‐Atf5‐CMV (ATF5‐OE) viral particles. After 3‐weeks transfection, ATF5 was overexpressed in mature oocytes of ATF5‐OE mice. B) Ovary H&E staining in CON and ATF5‐OE mice. Scale bar 200 µm, 50 µm (n = 5). The primary, second and mature oocytes were quantified per ovary section. C) Mature oocytes were collected in the fallopian tubes. Stars indicate the abnormal oocytes, and scale bar 50 µm. D, E) Immunocytochemical staining of mitochondria (MitoSpy; green color) and mitochondrial membrane potential (TMRE, red color) in mature oocytes isolated in CON and ATF5‐OE females. Intensity was quantified by Image J, and normalized by MitoSpy/TMRE. Scar bar MitoSpy, 200 µm; bright field, 150 µm; TMRE, 80 µm. Six mature oocytes were used in the analyses. F) mtDNA content in mature oocytes (n = 5). G) The percentage of mtDNA heteroplasmy, and number of mutation sites from POLG‐immunoprecipitate in mature oocytes (n = 5). H, I) Circular plot of the mitochondrial genome shows the genome annotation on the outer circle. Green inner circles and red outside cycles show the genomic locations and the relative frequencies of mtDNA mutations from POLG‐immunoprecipitants of mature oocytes from CON and ATF5‐OE females (n = 5) (H). Relative fold changes of mtDNA heteroplasmy from POLG‐immunoprecipitants between CON and ATF5‐OE mature oocytes were mapped (I). The heteroplasmic change was ATF5‐OE compared with controls. Regions corresponding to the different mtDNA genes (green, protein coding genes; pink, rRNA; red, tRNA; blue, non‐coding regions) (n = 5). J) Number of mtDNA mutation sites in mtDNA D‐Loop and protein coding‐genes in mtDNA from POLG‐immunoprecipitants (n = 5). Data are presented as mean ± s.e.m. *P < 0.05, **P < 0.01, ***P < 0.001, and ****P < 0.0001; unpaired two‐tail Student's t test was used in analyses.

To validate the ATF5‐POLG axis in driving mtDNA heroplasmy in mature oocytes, we overexpressed ATF5 protein in mature oocytes via administration of AAV09‐ATF5‐CMV viral particles to 3‐month old female mice (**Figure** **4**A). After 3‐weeks transfection, ATF5 overexpression highly reduced oocyte maturation, mitochondrial biogenesis and membrane poteintals (Figure 4B–E; Figure S4A,B, Supporting Information). The mtDNA content was not altered, while ATF5 activation substantially increased its binding affinity to POLG protein (Figure 4F; Figure S4C, Supporting Information) and promoted POLG protein binding to mutated mtDNA in driving mtDNA heteroplasmy in mature oocytes (Figure 4G–J), showing ATF5‐POLG complex alternatively activates heteroplasmic mtDNA replications in mature oocytes.

### 2.4 AMPK Inactivation Dysregulates Oocyte Maturation and mtDNA Heteroplasmy

**Figure 5:**
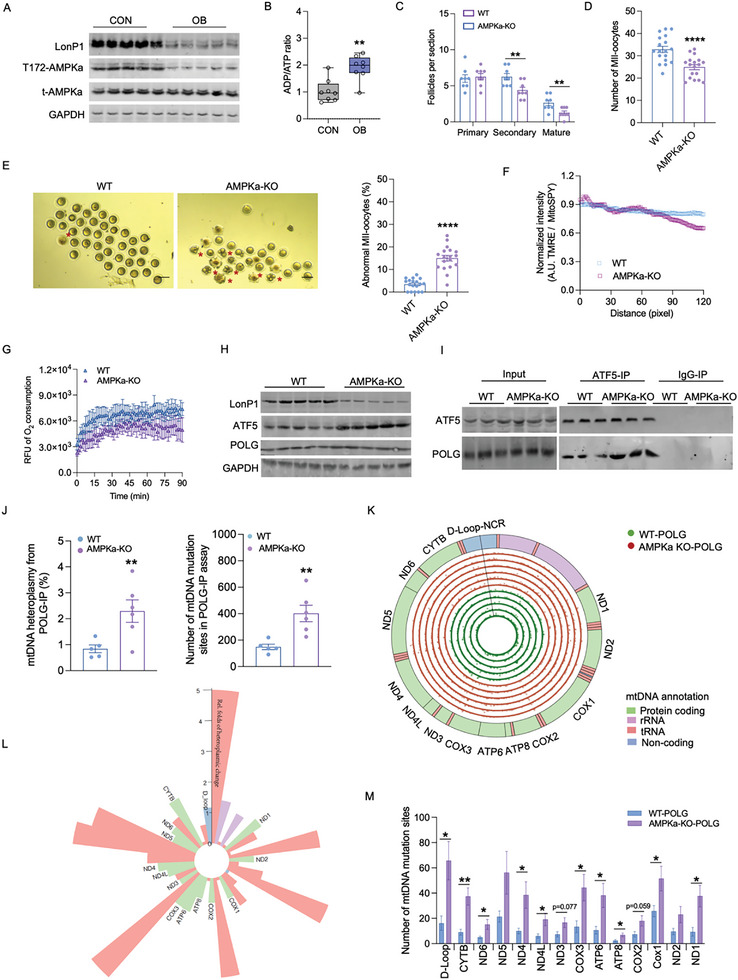
AMPK inactivation impairs oocyte maturation and ATF5‐POLG‐mtSNVs axis. A) Immunoblotting of LonP1, AMPKa and phosphorylated AMPKa at Thr172 in mature oocytes of control and obese females. GAPDH was used as a loading control (n = 5). B) The ADP/ATP ratio in mature oocytes (n = 8). C) The primary, secondary and mature follicles per section of ovaries (n = 8). D, E) The total number (D) and abnormal MII oocytes (E) were collected in the fallopian tube in wild type (WT) and Prkaa1‐knockout (AMPKa1‐KO) mice (n = 18). Star indicates mature oocytes with abnormal morphology. F) Immunostaining of MitoSpy (green color) and TMRE (red color) in mature oocytes, and fluorescent intensity was qualified by ImageJ and normalized by MitoSpy/TMRE. G) Oxygen consumption was measured in WT and AMPKa1‐KO oocytes. H) Immunoblotting of LonP1, ATF5 and POLG proteins in mature oocytes. GAPDH was used as a loading control (n = 5). I) Co‐immunoprecipitation of ATF5 in measuring POLG in mature oocytes. ATF5 and POLG were immunoprecipitated followed with SDS‐PAGE separation and measured by immunoblotting. IgG was used as a negative control in immunoprecipitation (n = 5). J) The mtDNA heteroplasmy and total mutation sites derived from POLG immunoprecipitants in mature oocytes (n = 6). K) Circular plot of the mitochondrial genome shows the genome annotation on the outer circle. The six green inner circles and six red outside cycles show the genomic locations and the relative frequencies of mtDNA mutations in POLG immunoprecipitants of mature oocytes of WT (green inner cycle) and AMPK‐KO mice (red outside cycle). L) Relative fold changes of mtDNA heteroplasmy in mature oocytes of WT and AMPK‐KO mice. The heteroplasmic change was AMPK‐KO compared with WT. Regions corresponding to the different mtDNA genes (green, protein coding genes; pink, rRNA; red, tRNA; blue, non‐coding regions) (n = 6). M) Number of mutation sites in mtDNA D‐loop and protein coding genes in mature oocytes (n = 6). Data are presented as mean ± s.e.m. *P < 0.05, **P < 0.01, ***P < 0.001, and ****P < 0.0001; unpaired two‐tail Student's t test and two‐way ANOVA with Bonferroni post hoc were used in analyses.

Next, we examined the activity of LonP1, a dominant ATP‐dependent protease responsible for degrading ATF5 and suppressing ATF5‐induced UPR^mt^. Intriguingly, while ATF5 and UPR^mt^ were highly activated in OB oocytes (Figure 3A; Figure S3A–C, Supporting Information), LonP1 protein was significantly reduced (**Figure** **5**A). We also observed a marked inhibition of AMPK activity in OB oocytes (Figure 5A; Figure S5A, Supporting Information). Given that LonP1 activity primarily relies on ATP/ADP ratio that was notably inhibited by OB in mature oocytes (Figure 5B), and AMPK is critical to regulate energy homeostasis,^[^ ^18^ ^]^ we further hypothesized that AMPK inactivation mediates the OB‐induced dysregulation of the ATF5‐POLG‐mtSNVs axis, ultimately leading to impaired mitochondrial OXPHOS and oocyte maturation.

To test, we conditionally knockout AMPKa1 (gene: Prkaa1), a dominant catalytic subunit of AMPK, in oocytes via intraperitoneal injection of tamoxifen in Prkaa1^fl/fl^/creER ^(+/‐)^ mice (AMPKa1‐KO) (Figure S5B, Supporting Information). AMPKa1 deletion not only reduced the formation of secondary and antral follicles, but also reduced the number of mature oocytes in oviducts (Figure 5C,D; Figure S5C, Supporting Information). Furthermore, oocyte quality was also compromised, as evidenced by reduced oocyte viability, mitochondrial membrane potential and oxygen consumption rate (Figure 5E–G; Figure S5D,E, Supporting Information). In addition, AMPKa1 knockout reduced ATP production and LonP1 content in mature oocytes (Figure 5H; Figure S5F,G, Supporting Information), leading to the accumulation of ATF5 protein (Figure 5H; Figure S5G, Supporting Information). Aligned with the impaired quality of OB oocytes, AMPKa1 knockout substantially increased the binding affinity of POLG to ATF5, and elevated the POLG attachment to mutated mtDNA in mature oocytes, including the number of A>C, C>T, C>G and C>A mutates in D‐loops (Figure 5I–M; Figure S5H,I, Supporting Information), showing AMPK inactivation resulted in dysregulation of LonP1‐ATF5‐POLG axis and impairment of oocyte quality.

### 2.5 AMPK Mediates Obesity‐Induced Dysregulation of LonP1‐ATF5‐POLG Axis

**Figure 6:**
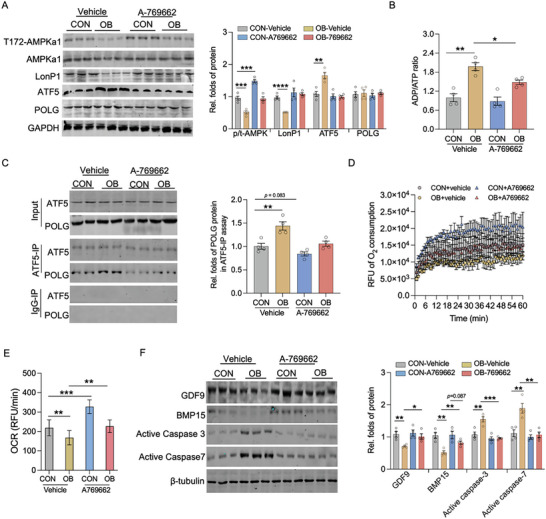
AMPK mediates the dysregulation in LonP1‐ATF5‐POLG axis and oocyte maturation due to obesity. A) Oocytes were isolated in control (CON) and obese females (OB), and treated AMPKa agonists A‐769662 in vitro. Immunoblotting of AMPKa, AMPKa phosphorylation at Thr‐172, LonP1, ATF5 and POLG proteins in mature oocytes. GAPDH was used as loading controls (n = 4). B) ADP/ATP ratio in mature oocytes (n = 4). C) Co‐immunoprecipitation of ATF5 in measuring POLG in mature oocytes. ATF5 and POLG were immunoprecipitated followed with SDS‐PAGE separation and measured in immunoblotting. IgG was used as a negative control in immunoprecipitation (n = 4). D, E) Oxygen consumption was measured in mature oocytes supplemented with A‐769662. F) Immunoblotting of GDF9, BMP15, active‐caspase3 and active‐caspase7 proteins in mature oocytes. β‐tubulin was used as a loading control (n = 4). Data are presented as mean ± s.e.m. *P < 0.05, **P < 0.01, ***P < 0.001, and ****P < 0.0001; Two‐way ANOVA with Bonferroni post hoc was used in data analysis; obesity and AMPKa activator were as main factors in the analysis.

To further uncover the AMPK roles in regulating ATF5‐POLG axis and oocyte quality in OB oocytes, we treated OB oocytes with AMPK‐specific agonists A‐769662 and metformin to activate AMPK activity in vitro (**Figure** **6**A; Figure S6A, Supporting Information). Compared to normal oocytes, OB oocytes exhibited a significant reduction of ATP production, LonP1 content and an increase of ATF5 protein in mature oocytes (Figure 6A,B; Figure S6A,B, Supporting Information). However, these alterations were substantially blocked by the addition of AMPK agonists (Figure 6A,B; Figure S6A,B, Supporting Information). Consistently, treatment with AMPK activators significantly reduced the binding affinity of ATF5 protein to POLG in OB oocytes (Figure 6C; Figure S6C, Supporting Information), and elevated the oxygen consumption (Figure 6D,E; Figure S6D,E, Supporting Information) and the levels of oocyte maturation markers (Figure 6F; Figure S6F, Supporting Information).

**Figure 7:**
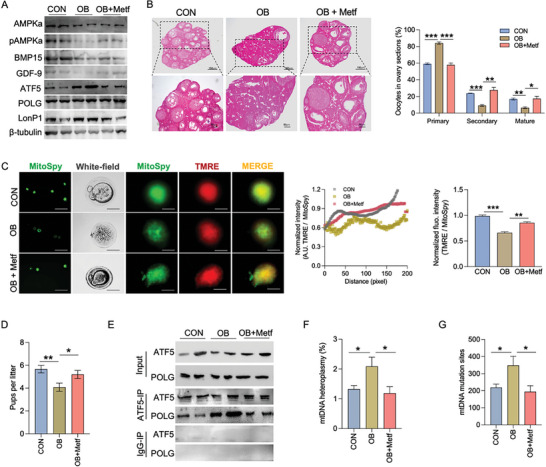
AMPK activation improves oocyte maturation and limits mtDNA heteroplasmy in obese females. A) Obese females were fed metformin in drinking water for 4‐weeks (OB+Metf). Immunoblotting of AMPKa, AMPKa phosphorylation at Thr‐172, BMP15, GDF‐9, ATF5, POLG and LonP1 proteins in mature oocytes of control (CON), obese (OB) and OB+Metf females. The β‐tubulin was used as a loading control (n = 5). B) H&E staining in ovary. The primary, secondary and mature oocytes were quantified in each ovary section (n = 5). C) Immunostaining of MitoSpy (green color) and TMRE (red color) in mature oocytes. Fluorescent intensity was qualified by ImageJ and normalized by MitoSpy/TMRE. Five mature oocytes were used in the analyses. Scar bar MitoSpy, 200 µm; bright field, 150 µm; TMRE, 80 µm. D) Number of pups per litter born from female mice. E) Co‐immunoprecipitation of ATF5 in measuring POLG in mature oocytes. ATF5 and POLG were immunoprecipitated followed with SDS‐PAGE separation and measured in immunoblotting. IgG was used as a negative control in immunoprecipitation. F) Percentage of mtDNA heteroplasmy and total mutation sites in mature oocytes from mtDNA‐sequencing (n = 5). Data are presented as mean ± s.e.m. *P < 0.05, **P < 0.01, ***P < 0.001, and ****P < 0.0001; one‐way ANOVA was used in analysis.

Furthermore, we fed obese females with metformin for 4‐weeks to activate AMPK in oocytes in vivo (**Figure** **7**A). In OB females, supplementation of AMPK agonist not only improved oocyte maturation and ovulation (Figure 7B; Figure S7A, Supporting Information), but also increased mitochondrial density and membrane potential (Figure 7C; Figure S7B, Supporting Information). Aligned with an improved oocyte quantity and quality, AMPK activation highly alleviated the detrimental impacts of female obesity on litter size (Figure 7D). Besides, activator addition reduced the binding affinity of ATF5 to POLG protein, attributing to the reduced mtDNA heteroplasmy and mtSNVs in D‐Loop and protein‐coding genes in mature oocytes (Figure 7E–G; Figure S7C, Supporting Information). Taken together, these data show that AMPK inactivation plays a vital role in mediating the dysregulation of LonP1‐ATF5‐POLG axis and oocyte maturation in OB females.

## 3 Discussion

**Figure 8:**
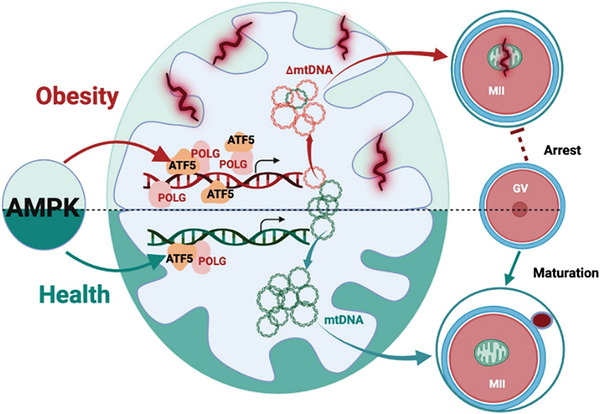
Diagram summaries that obesity induces AMPK inactivation, lower mtDNA quantity and quality in mature oocytes, attributing to impaired mitochondrial energetics and female fertility. AMPK inactivation increases binding affinity of ATF5‐POLG protein complex to D‐loop and protein‐coding regions of mutated mtDNA, leading to replication of heteroplasmic mtDNA, impaired mitochondrial biogenesis and oocyte quality. AMPK activation blocks the detrimental impacts of OB by preventing ATF5‐POLG protein recruitment, benefiting oocyte maturation and fertility.

Obesity in women is associated with increased risks of ovulation disorders, reduced fertility and disrupted offspring growth.^[^ ^24^ ^]^ Meta‐analyses consistently showed lower oocyte yields, higher miscarriage rates and reduced birth rates in obese women.^[^ ^25^ ^]^ Even with the utilization of assisted reproductive technologies, the live birth rates of obese women are approximately 50% lower than their non‐obese counterparts, with a 2–4% reduction in implantation rates for each one unit increase in body mass index.^[^ ^26^ ^]^ Despite compelling pathological evidence demonstrating that obesity diminishes both the quality and quantity of oocytes,^[^ ^3^, ^25^, ^27^ ^]^ the underlying mechanisms remain elusive. Oocyte maturation involves substantial increase in size and biochemical transformations, accompanied with enormous expansion of mtDNA.^[^ ^6^, ^8^ ^]^ Our study revealed that obesity hindered oocyte maturation and female fertility, while reducing mtDNA content and increasing mtDNA mutations in D‐loop and protein‐coding genes, contributing to impaired mitochondrial OXPHOS activity. Furthermore, we identified AMPK inactivation as a key factor mediating the impacts of obesity on mitochondrial biogenesis, mtDNA heteroplasmy and oocyte maturation, which involves the dysregulation of the LonP1‐ATF5‐POLG protein axis. Remarkably, such impairment can be effectively prevented using AMPK activators, suggesting the high potential of AMPK as a pharmaceutical target for enhancing mtDNA fidelity, oocyte maturation and female fertility of obese women (**Figure** **8** for a summary).

Mitochondrial numbers and mtDNA content massively expand during oocyte maturation.^[^ ^8^, ^22^ ^]^ After ovulation, oocytes lose connection with cumulus cells and heavily rely on their own mitochondria to provide the energy required for early embryonic growth.^[^ ^28^ ^]^ Previous studies showed that metabolic disorders associated with aging, obesity and diabetes can induce mitochondrial dysfunction in mature oocytes.^[^ ^12^, ^29^ ^]^ Our data align with these observations, showing that obesity not only reduces mitochondrial density but also disrupts mitochondrial membrane potential. As BMP‐15 and GDF‐9 proteins are robust indicators of high‐quality oogenesis and folliculogenesis,^[^ ^21^ ^]^ the reduced expression of GDF‐9 and BMP‐15 proteins, along with diminished cellular oxygen consumption, further supported impaired oocyte maturation and the presence of mitochondrial energetic dysfunctions in obese females. Additionally, mitochondrial fission regulator DRP1 is critical for reducing reactive oxygen species and removing dysfunctional mitochondria in oocytes,^[^ ^30^ ^]^ which was lower in mature oocytes of obese females, emphasizing the significant accumulation of damaged mitochondria and unfolded protein responses.

The mtDNA content and heteroplasmy play crucial roles in regulating oocyte quality^[^ ^28^ ^]^ Mature oocytes from patients with ovarian dystrophy contain approximately 60% less mtDNA content.^[^ ^31^ ^]^ Meanwhile, mtDNA heteroplasmy can induce follicular atresia, leading to fewer ovulating oocytes in both human and mice.^[^ ^28^, ^32^ ^]^ Maternal aging and diabetes increase mtDNA mutations and oocyte aging,^[^ 3, 12, 29 but the impacts of obesity on mtDNA heteroplasmy remain largely unexplored. We found that obesity not only decreased mtDNA content, but also increased mtDNA heteroplasmy in mature oocytes. The mtDNA mutations were predominantly found in D‐loop and protein coding genes, potentially attributing to the notion of reduced mtDNA transcriptional activity in obese females. Moreover, mutated mtDNA may also preferentially replicate and expand during the late oocyte development, which could be transmitted to embryos to affect their development.^[^ ^11b^ ^]^ Since somatic tissues and organs in offspring have very limited capacity to clear mutated mtDNAs, mtDNA heteroplasmy in oocytes predisposes offspring to mitochondria‐related metabolic disorders, such as obesity and type 2 diabetes. Of note, due to the limited content of mtDNA mutations detected, the cause of UPR^mt^ activation in obese oocytes could also be driven by the impaired mitochondrial OXPHOS, low mtDNA content and other cellular dysfunctions.

ATF5 has emerged as a central player in UPR^mt^, which can promote mtSNVs production.^[^ ^14^, ^15^, ^17^ ^]^ Under normal conditions, the majority of ATF5 can be imported into mitochondria and subsequently degraded by LonP1 protease. However, under metabolic dysfunctions or stress, such as starvation, ATF5 accumulates in dysfunctional mitochondria and induces the replication of heteroplasmic mtDNA.^[^ ^14^ ^]^ Recently, ATF5 is shown to mediate the replication of heteroplasmic mtDNA with m.3243A > G, attributing to mitochondrial dysfunctions and osteoporosis in patients.^[^ ^15^ ^]^ In this study, we observed the ATF5 accumulation within mitochondria, which was associated with elevated UPR^mt^ in oocytes of OB females. Moreover, obesity elicited a higher binding between POLG and ATF5 protein, suggesting that the ATF5‐POLG complex might drive mutated mtDNA replication. Our data are consistent with recent observation in *C. elegans*, where ATF5 homologs, ATFS‐1, can promote defective mtDNA expansion, leading to the accumulation of defective mitochondria.^[^ ^14^, ^17^ ^]^ These data support the critical role of ATF5‐POLG axis potentially in promoting mutated mtDNA accumulation due to obesity.

The dysregulation of the ATF5‐POLG‐mtSNVs axis in obese oocytes prompted us to investigate the role of AMPK in mediating these effects. Although AMPK inactivation has been shown to impair germinal vesicle break down and oocyte maturation,^[^ ^19^ ^]^ its role in mediating the effects of obesity on oocyte maturation and mtDNA heteroplasmy has not been explored. Given the dependence of LonP1 activity on the ATP/ADP ratio,^[^ ^16a^ ^]^ and the inhibition of LonP1 expression in obese oocytes, we assessed the roles of AMPK in mediating the dysregulation of ATF5‐POLG axis and oocyte quality. Conditionally knockout of AMPKa1 in oocytes resulted in reduced antral follicle formation and a lower number of mature oocytes. These effects were accompanied by reduced ATP production and LonP1 activity in mature oocytes, which contributed to the accumulation of ATF5 protein and an increase in POLG‐bound mutated mtDNA, underscoring the essential role of AMPK in regulating the ATF5‐POLG axis and oocyte quality. Consistently, when AMPK was activated in vitro or in vivo, it could substantially limit the ATF5‐POLG protein binding and mtDNA heteroplasmy, improving the oocyte maturation and fertility of obese females. Recently, LonP1 inactivation was identified to increase UPR^mt^ and impair oocyte maturation,^[^ ^33^ ^]^ further supporting that AMPK regulates mitochondrial biogenesis and mtDNA heteroplasmy in oocytes through LonP1‐ATF5‐POLG pathway.

## 4 Conclusion

This study unveils the multifaced impacts of obesity on oocyte maturation and energetic dysfunction through the LonP1‐ATF5‐POLG axis. AMPK emerges as a key mediator in the dysregulation and highlights its potential as a therapeutic target to mitigate the adverse effects of obesity on mitochondrial biogenesis and mtDNA heteroplasmy, oocyte maturation and quality, offering promising avenues to improve reproductive and health outcomes of both mothers and their offspring.

## 5 Experimental Section

### Animals and Mature Oocyte Harvesting

Wild‐type female C57BL/6J mice (The Jackson Laboratory, Bar Harbor, ME) at 2 months of age were fed ad *libitum* either a standard chow diet (10% energy from fat; D12450H, Research Diets, NJ, USA) or an obesogenic diet (OB; 60% energy from fat; D12492, Research Diet) for 8 weeks after weaning. MII mature oocytes were collected by intraperitoneal injection of 7.5 IU of pregnant mare serum gonadotropin (PMSG), followed by an additional injection of 7.5 IU human chorionic gonadotropin (hCG) after 48 h. After 12 h following hCG injection, cumulus oocyte complexes were isolated from the ampulla of fallopian tube. MII oocytes were stripped and collected after brief exposure to 400 IU/ml hyaluronidase (H4272; Sigma). Each set of 80–100 MII oocytes was further cultured in M2 medium (M7167; Sigma) supplemented with 1 µ*M* A‐769662 (#SML2578; Sigma) or 50 µ*M* metformin (#1 396 309; Sigma) for 24 h.^[^ ^34^ ^]^ To determine the impacts of AMPK activator on oocyte quality in vivo, OB females were treated with 2 mg ml^−1^ metformin (#M58670; Acmec, Nanjing) in drinking water for 4 weeks prior to mature oocyte isolation.^[^ ^35^ ^]^

To achieve AMPKa1 (gene: *Prkaa1*) conditional knockout, ER‐Cre mice (#0 04847; Jackson lab) were crossed with Prkaa1^flox/flox^ (#01 4141) to obtain creER^−/−^/Prkaa1^fl/fl^ and creER^+/−^/Prkaa1^fl/fl^ mice. To induce AMPKa1 knockout, crossbred creER^+/−^/Prkaa1^fl/fl^ female mice at 3‐month‐age were intraperitoneally injected with tamoxifen (75 µg g^−1^ of body weight) following oocyte harvesting. Due to the energy dysfunction associated with AMPKa1 ablation and occasional sudden mouse death within 3‐weeks of tamoxifen administration, AMPKa1 knockout mice were not subjected to further dietary treatments.

To generate adeno‐associated viruses carrying AAV‐ATF5, the DNA fragment coding for Atf5 was subcloned into a pAAV cloning plasmid (OBIO biology, Shanghai, China). To produce AAV viral particles with serotype AAV9, pAAV‐Atf5 vector was co‐transfected into 293T cells with adenoviral helper genes and AAV9 cap genes for producing recombinant AAV9‐Atf5‐CMV viral particles, which have high transfection efficiency in ovary.^[^ ^36^ ^]^ Viral particles were purified by ultracentrifugation at 350000 × g in an iodixanol gradient concentration using Amicon Ultra‐15, and the titer of virus was 1 × 10^13^ genome copies ml^−1^. Approximate 1 × 10^11^ viral particles were injected into the tail vein of 3‐month old mice, and ovaries were collected after 3‐weeks of administration. All mice were housed at 22˚C, 12 h light: 12 h dark cycle, and euthanized by CO_2_. The animal facility was accredited by the Association for Assessment and Accreditation of Laboratory Animal Care (AAALAC), and all animal studies were conducted according to the protocol approved by the Institute of Animal Care and Use Committee (IACUC) at Washington State University (ASAF#6704; Pullman, WA, USA) and Nanjing Agricultural University (#20 221 206 231; Nanjing, Jiangsu, China).

### Real‐Time qPCR Analyses

Total RNA was isolated using TRIzol reagent (Invitrogen, NY, USA) according to manufacturer's guidelines.^[^ ^24^, ^37^ ^]^ The mRNA was converted to cDNA using an iScript cDNA synthesis kit (Bio‐Rad, CA, USA). Real‐time qPCR (IQ5, Bio‐Rad) was performed using the iQTM SYBR Green Supermix (Bio‐Rad) system, and data were collected with the Bio‐Rad CFX manager 3.1 software (CFX Connect). Primer sequences were provided in SI Appendix Table S1 (Supporting Information).

### Immunoblotting

Approximate 50 oocytes were used for immunoblotting analyses. Protein samples were separated by 10% polyacrylamide gels and transferred onto a nitrocellulose membrane.^[^ ^24^, ^37^ ^]^ Membrane were probed using primary antibodies, including BMP‐15 (#18 982; Proteintech), GDF‐9 (af739; Novus), Caspase‐7 (sc‐56063; Snta‐Cruz), Caspase‐3 (#9662; Cell Signaling), β‐tubulin (#179 513; Abcam), DRP1 (#8570; Cell Signaling), p‐DRP1 (#3455; Cell Signaling), VDAC (#4866; Cell Signaling), TOM20 (sc‐17764; Santa Cruz), ATF5 (ab184923; Abcam), LonP1 (#15 440; Proteintech), POLG (ab128899; Abcam), HSP70 (#4872; Cell Signaling), AMPKa (D63G4; Cell Signaling), p‐AMPKa (#2535; Cell Signaling), GAPDH (#97 166; Cell Signaling). The secondary antibodies were purchased from LI‐COR bioscience, including anti‐mouse IRDye 650 and anti‐rabbit IRDye 800CW. Signals were detected by an infrared imaging system (Odyssey, LI‐COR Bioscience), and protein intensity was quantified using Image Studio Lite 5.2 (LI‐COR Bioscience).

For isolating crude mitochondrial and cytosol fractions, ≈ 80 oocytes were homogenized in ice cold lysis buffer (10 mM HEPES, 0.1 mM EGTA, 250 mM sucrose, pH 7.2) supplemented with 2% BSA. Homogenates were centrifuged at 8500 g for 15 min to collect pellets containing mitochondria, nuclei and cell debris. Pellets were further suspended and centrifuged at 700 g for 10 min to separate mitochondria from nuclei and cell debris. Supernatant was centrifuged at 8500 g for 10 min to pellet crude mitochondria for immunoblotting analyses.

### Immunocytochemical and H&E Staining

Ovaries were fixed in 4% paraformaldehyde (PFA) and embedded in paraffin. Tissue sections were sliced and subjected to immunocytochemical and H&E staining. Immunostaining included the detection of mitochondria density using MitoSpy Green (#424 806; Biolegend) and target proteins using primary antibodies against caspase‐7 (sc‐56063; Snta‐Cruz) and HSP70 (#4872; Cell Signaling). Live oocytes were detected based on the mitochondrial membrane potential using TMRE (#115 532; Sigma). Images were obtained by an EVOS XL Core imaging system (Mil Creek, WA, USA), and fluorescent intensity was quantified by ImageJ.

### Co‐Immunoprecipitation Assay

Co‐immunoprecipitation analysis was conducted as previously described.^[^ ^24^, ^37^ ^]^ Cell lysates containing protease inhibitors (#87 786; Thermo Fisher) were precleared with protein A magnetic beads (#73 778; Cell Signaling) at 4˚C for 1 h. Primary antibody ATF5 (ab184923; Abcam) was added to lysates containing 500 µg total protein and rotated overnight at 4 °C. Protein A beads were added to the pre‐cleaned solution and gently rotated at room temperature for 4 h. Beads were washed with cold PBS three times and collect for western blotting.

### Oxygen Consumption Rate

Extracellular oxygen consumption rate was measured according to an assay protocol (ab197243; Abcam).^[^ ^24^ ^]^ Oocytes were cultured in M2 medium at 37˚C. Mineral oil was added to limit the diffusion of oxygen into assay medium. Fluorescence intensity was measured by a time‐lapse microreader (Biotek Synergy H1, Agilent) at 360 nm excitation and 680 nm emission.

### mtDNA Sequencing and Analyses

Oocytes were collected and used for mtDNA isolation following manufacturer's guidelines (ab65321; Abcam). Harvested mtDNA were detected by the agarose gel electrophoresis and quantified by QubitR 2.0 fluorometer (Thermo Fisher). Sequencing libraries were generated using a NEBNextR Ultra DNA library Pre Kit from Illumina (NEB, USA) following manufacture's protocol, and index codes were added to each sample. Libraries were analyzed for size distribution by Agilent2100 Bioanalyzer and quantified by qPCR. The libraries were sequenced using Illumina PE150. Trimmomatic‐0.38 was used for data filter and data cleaning. Bwa‐0.7.12 was employed for data mapping to the reference sequence (accession NC_0 05089.1), and GATK‐3.8‐0, Samtools‐1.9 and Varscan‐v2.4.3 were used for SNP/Indel detection.^[^ ^38^ ^]^ The mutations were not included if their minimal heteroplasmic fraction was less than 1% per strand.

### mtDNA Copy Number

Total DNA was extracted from oocytes using phenol/chloroform/isoamyl alcohol (25:24:1) followed by 70% ethanol precipitation. The content of mtDNA was calculated using qPCR by measuring the threshold cycle ratio of mitochondrial gene *Cox1* versus nuclear gene *Gtl2*. Primer sequences were provided in SI Appendix Table S1 (Supporting Information).

### ATP / ADP Ratio Assay

ADP and ATP levels were detected according to the assay protocol (MAK135; Sigma, MO, USA). In the ADP/ATP assay, oocytes were added D‐luciferin and incubated for 1 min at room temperature. ATP content was determined by a luminescence plate reader. Oocytes were further incubated for 10 min followed by adding ADP reaction reagents. After incubation for 1 min, the luminescence was measured again.

### Statistical Analysis

Data were presented as mean ± s.e.m. All statistical analyses were performed using SAS version 9.4 (SAS Institute, NC, USA) and figures were prepared using GraphPad Prism7 (San Diego, CA, USA). Unpaired two‐tail Student's t test, one‐way and two‐way ANOVA with Bonferroni post hoc were used in data analysis. Two‐way ANOVA was used for analyzing two main factors: obesity and AMPK activators for data from in vitro oocyte studies. Significant differences were indicated as *P < 0.05, **P < 0.01, and *** *P* < 0.001.

## Conflict of Interest

The authors declare no conflict of interest.

## Author Contributions

Y.T.C., G.L.M. and Y.G. contributed equally to this work. Y.T.C., M.D. developed the concept, designed experiments, and Y.T.C., M.D. interpreted the data. Y.T.C., G.L.M, Y.G, X.D.L, Q.Y.Y., M.J.Z. conducted experiments and collected data. G.L.M., Y.T.C., and Y.G. analyzed data. Y.T.C., M.D. prepared the manuscript. Y.T.C., M.D., G.L.M., S.Y.M., M.J.Z. made revisions to the manuscript. All authors approved the final content. Y.T.C. and M.D. were the guarantors of this work and had full access to the data in the study and take responsibility for the integrity of the data and accuracy of the data analysis.

## Supporting information

Supporting Information

## Data Availability

The data that support the findings of this study are available in the supplementary material of this article.
