# Supplementary material for: AMPK Suppression Due to Obesity Drives Oocyte mtDNA Heteroplasmy via ATF5‐POLG Axis
Source: Adv Sci (Weinh). 2024 Mar 18;11(20):2307480. doi: 10.1002/advs.202307480 (PMC11132083; doi:10.1002/advs.202307480)
Supplement: Supplementary file 1 — Supporting Information [file ADVS-11-2307480-s001.pdf]

ADVANCED SCIENCE
Open Access

Supporting Information

for *Adv. Sci.*, DOI 10.1002/advs.202307480

AMPK Suppression Due to Obesity Drives Oocyte mtDNA Heteroplasmy via ATF5-POLG Axis

Yanting Chen, Guiling Ma, Yang Gai, Qiyuan Yang, Xiangdong Liu, Jeanene M. de Avila, Shengyong Mao, Mei-Jun Zhu and Min Du**

Supporting Information

Title AMPK suppression due to obesity drives oocyte mtDNA heteroplasmy via ATF5-POLG axis

Yanting Chen[*]*, Guiling Ma, Yang Gai, Qiyuan Yang, Xiangdong Liu, Jeanene M. de Avila, Shengyong Mao, Mei-Jun Zhu, Min Du*[*]

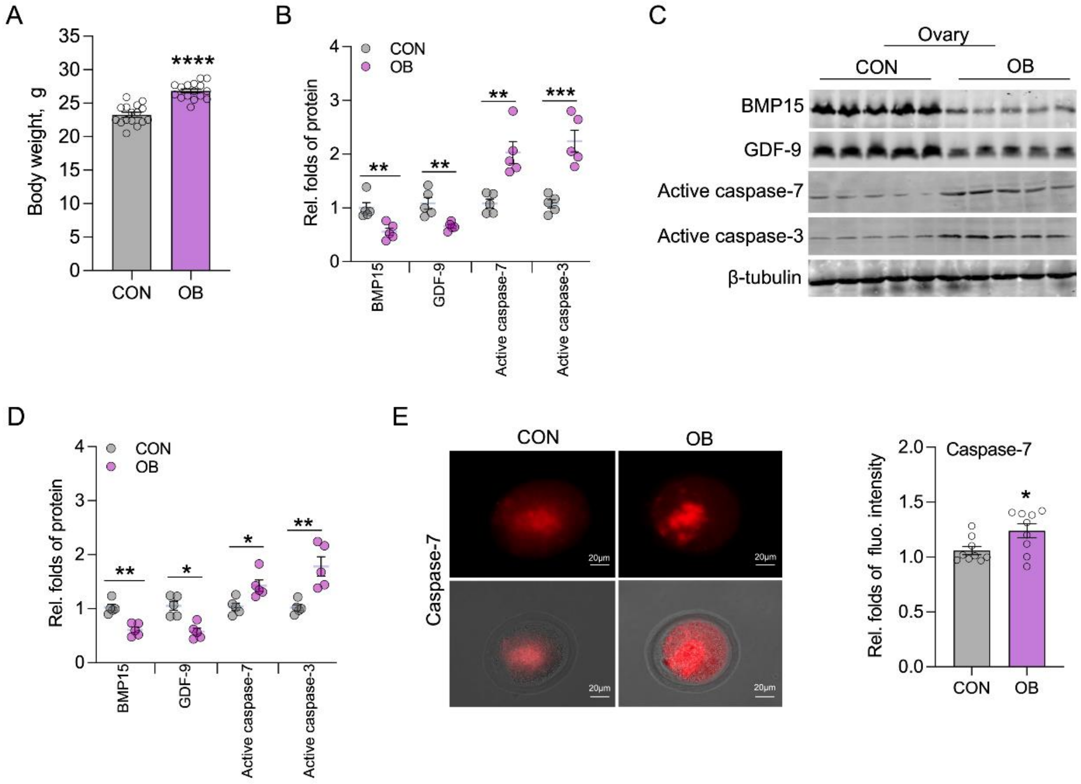

Figure S1. Obesity impairs follicle development and oocyte maturation. A) Body weight of female mice fed control (CON) and obesogenic diet (OB) (n = 16). B) Relative folds of protein contents in mature oocytes, including BMP15, GDF-9, active caspase-7 and caspase-3 (n = 5). C, D) Immunoblotting of BMP15, GDF-9, active caspase-7, and caspase-3 in ovaries. Β-Tubulin was used as a loading control (n = 5). E) Immunostaining of Caspase-7 in mature oocytes, and the quantified fluorescent intensity (n = 9). Scale bar 20 µm. Data are presented as mean ± s.e.m. **P* < 0.05, ***P* < 0.01, ****P* < 0.001, and *****P* < 0.0001; unpaired two-tail Student's t test was used in analyses.

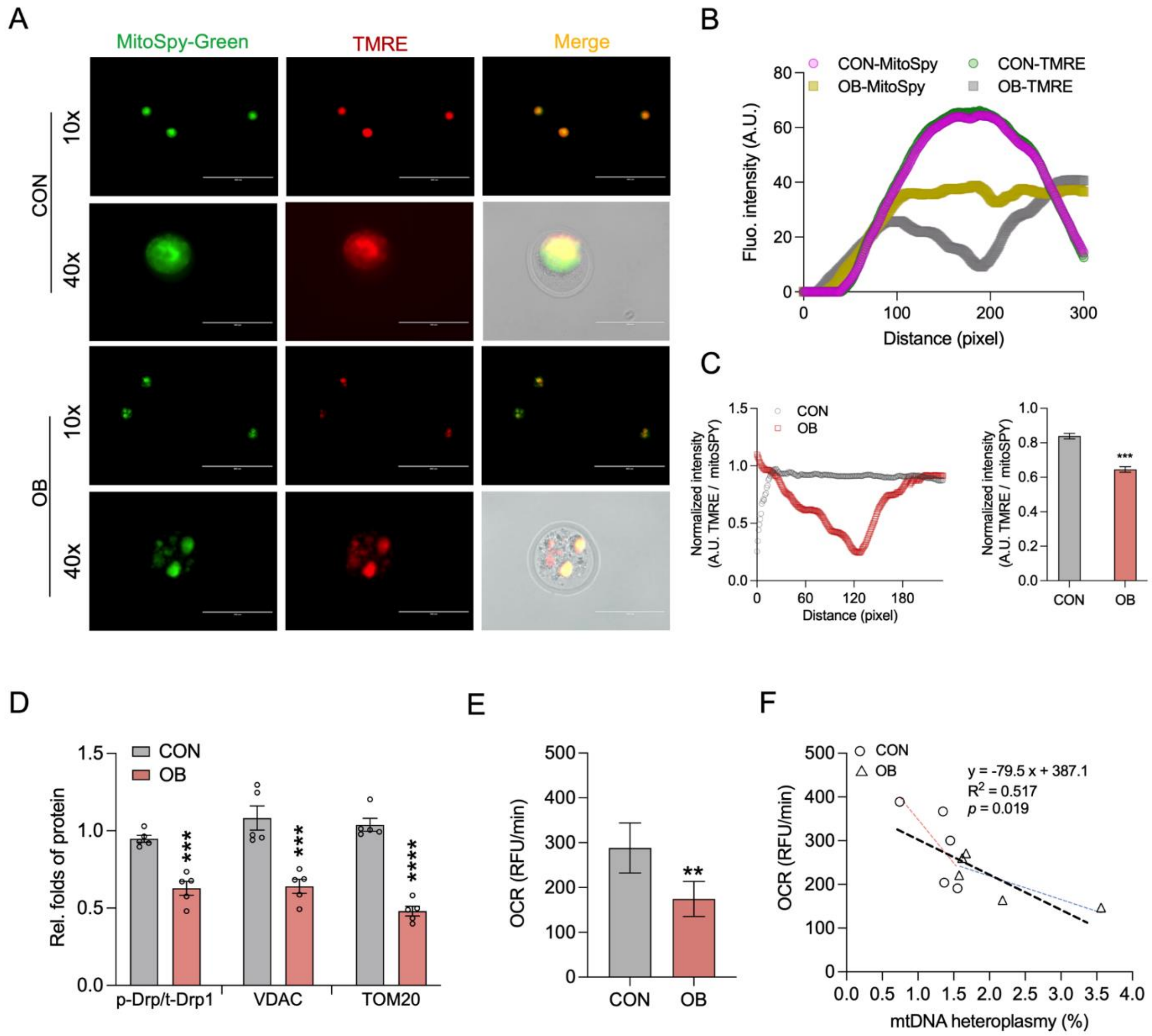

Figure S2. Obesity impairs follicle development and oocyte maturation. A, B, C) Immunocytochemical staining of mitochondria (MitoSpy; green color) and mitochondrial membrane potential (TMRE, red color) in mature oocytes isolated in control (CON) and obese (OB) females. Intensity was quantified by Image J and normalized by MitoSpy/TMRE. Five mature oocytes were used in the analyses. Scar bar 10 ×, 400 µm; 40 ×, 100 µm. D) Relative folds of protein contents in mature oocytes, including Drp1, Drp1-S616, VDAC and Tom20 (n = 5). E) Oxygen consumption was measured in mature oocytes. F) Linear regression analyses between mtDNA heteroplasmy and oxygen consumption rate in mature oocytes. Normal oocytes, pink dash line Y=-199.7x +548.4 R^2=0.49; OB oocytes, blue dash line Y=-53.8x +326.7 R^2=0.66. Data are presented as mean ± s.e.m. *P < 0.05, **P < 0.01, ***P < 0.001, and ****P < 0.0001; unpaired two-tail Student's t test was used in analyses.

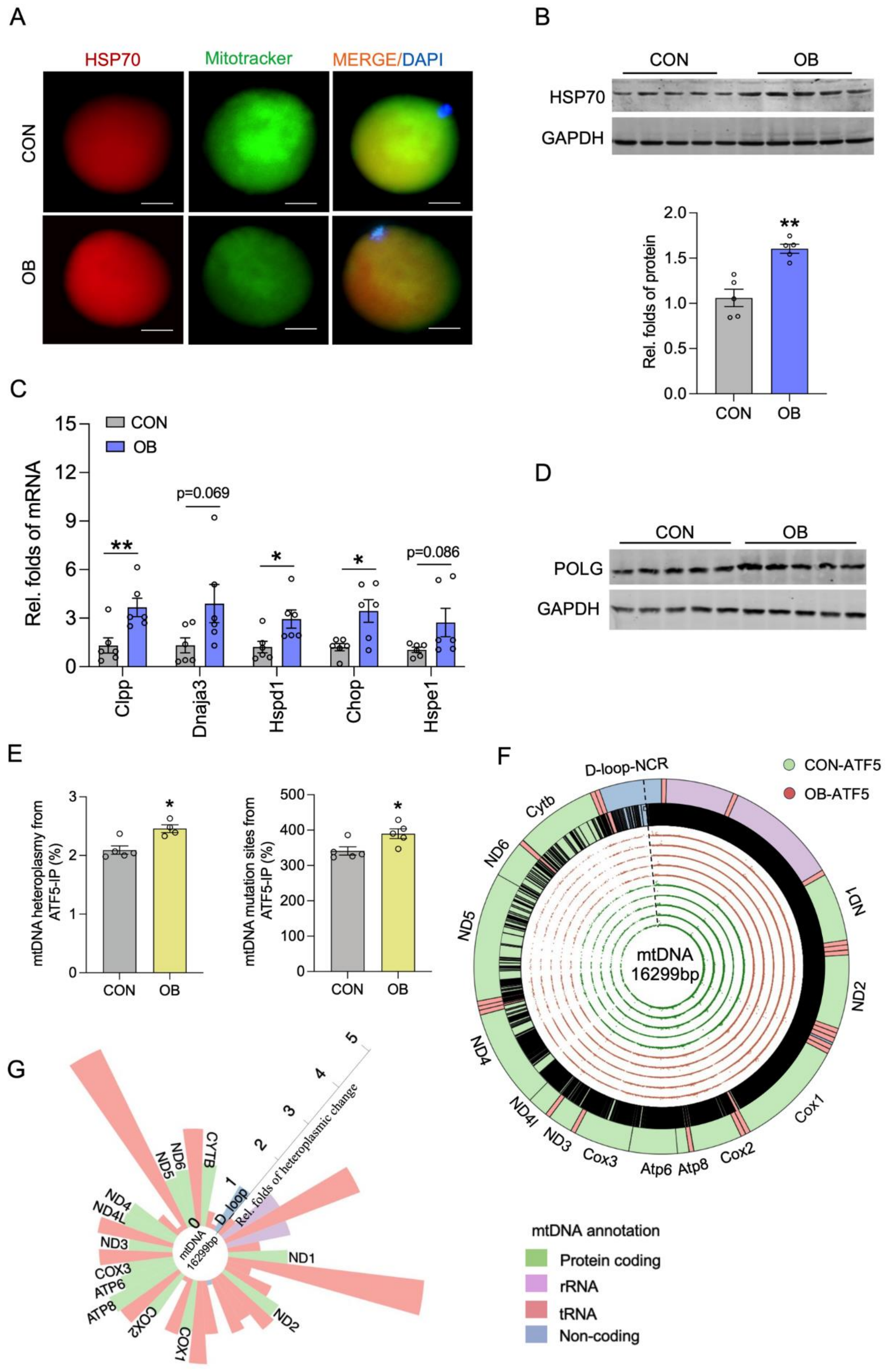

Figure S3. Excessive UPR^{mt} in mature oocytes of obese females. A) Immunostaining of mitospy (green color) and HSP70 (red color) in mature oocytes collected in both control (CON) and obese (OB) females. Blue is DAPI to indicate nucleus. Scale bar 20 µm. B) Immunoblotting of HSP70 in mature oocytes. GAPDH was used as a loading control (n = 5). C) The mRNA expression of mitochondrial unfolded protein response indicators, including

Clpp, *Dnaja3*, *Hspd1*, *Chop* and *Hspe1* (n = 6). mRNA was normalized to 18s rRNA. D) Immunoblotting of PLOG in mature oocytes. GAPDH was used as a loading control (n = 5). E) The percentage of mtDNA heteroplasmy and total mutation sites in ATF5-immunoprecipitation (n = 5). F, G) Circular plot of the mitochondrial genome shows the genome annotation on the outer circle. Five green inner circles and five red outside cycles show the genomic locations and the relative frequencies of mtDNA mutations from POLG immunoprecipitants in mature oocytes of CON (green inner cycle) and OB females (red outside cycle) (F). Relative fold changes of mtDNA heteroplasmy between CON and OB mature oocytes were mapped (G). The heteroplasmic change was OB compared with controls. Regions corresponding to the different mtDNA genes (green, protein coding genes; pink, rRNA; red, tRNA; blue, non-coding regions) (n = 5). Data are presented as mean ± s.e.m. **P* < 0.05, ***P* < 0.01, ****P* < 0.001, and *****P* < 0.0001; unpaired two-tail Student's t test was used in analyses.

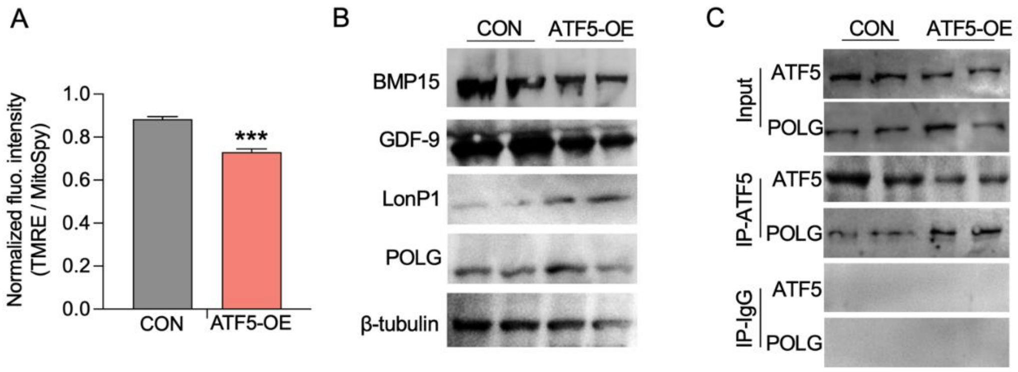

Figure S4. ATF5 activation impairs oocyte maturation and mtDNA heteroplasmy. A) Fluoresecent intensity in mature oocytes of control (CON) and ATF5-overexpressed (ATF5-OE) females was quantified by image J, and further normalized by MitoSpy/TMRE. B) Immunoblotting of BMP15, GDF-9, LonP1 and POLG in mature oocytes. β-tubulin was used as a loading control. C) Co-immunoprecipitation of ATF5 in measuring POLG in mature oocytes. ATF5 and POLG were immunoprecipitated followed with SDS-PAGE separation

and measured in immunoblotting. IgG was used as a negative control in immunoprecipitation. Data are presented as mean ± s.e.m. *$P < 0.05$, **$P < 0.01$, ***$P < 0.001$, and ****$P < 0.0001$; unpaired two-tail Student's t test was used in analyses.

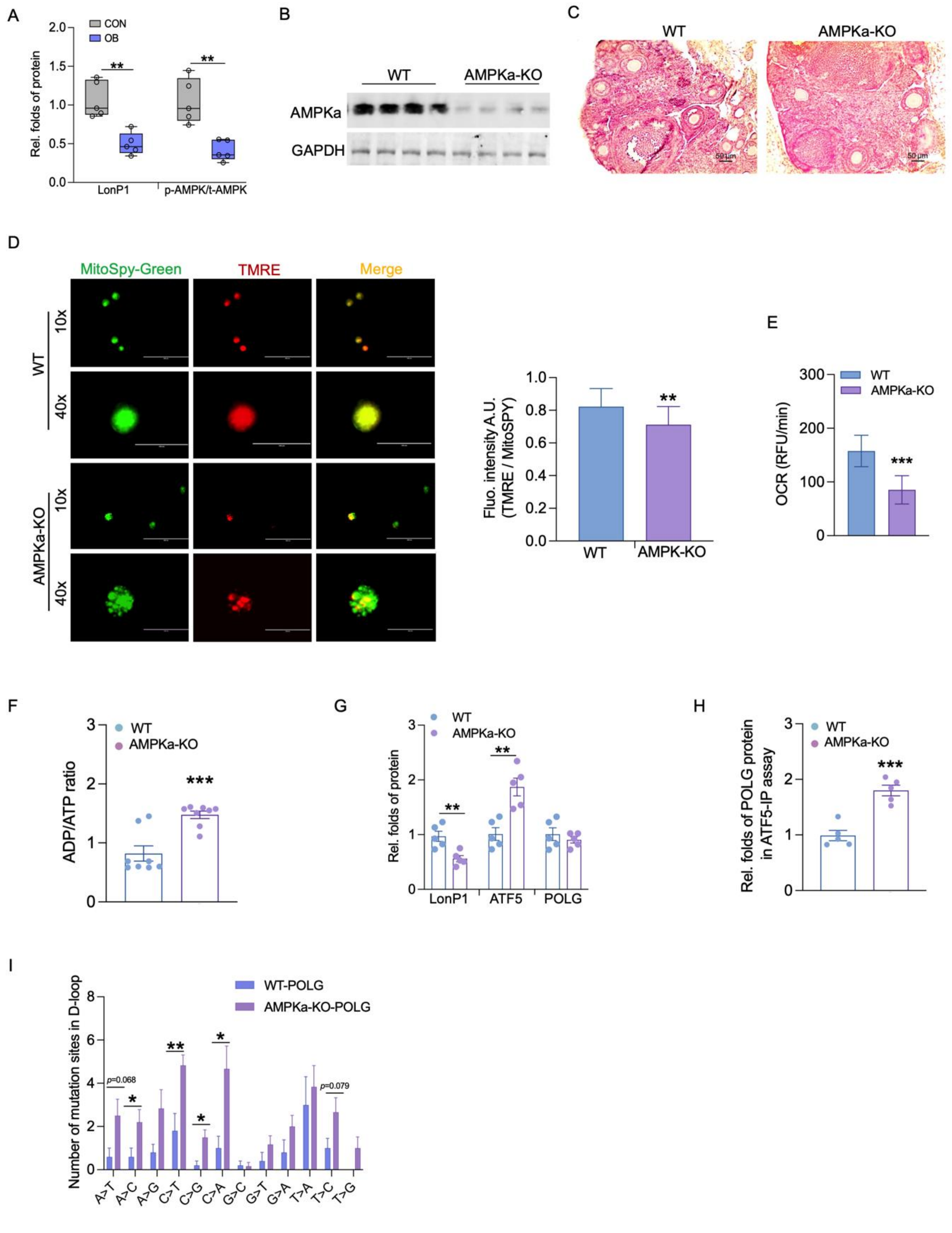

Figure S5. AMPK inactivation impairs mitochondrial energetics and oocyte maturation. A) Relative folds of protein content in mature oocytes, including LonP1, AMPKa and phosphorylated AMPKa Thr172 (n = 5). B) Immunoblotting of AMPKa1 in mature oocytes in wild type (WT) and Prkaa1-knockout (AMPKa1-KO) mice. GAPDH was used as a loading control. C) H&E staining of ovaries in WT and AMPKa1-KO mice. D) Immunostaining of MitoSpy (green color) and TMRE (red color) in mature oocytes, and fluorescent intensity was qualified by ImageJ and normalized by MitoSpy/TMRE. At least 10 mature oocytes were

used in the analyses. Scale bar 10×, 400 μm; 40×, 100 μm. E) Oxygen consumption was measured in WT and AMPKa1-KO oocytes. F) ADP/ATP ratio in mature oocytes (n = 8). G) Protein content of LonP1, ATF5 and POLG proteins in WT and AMPKa1-KO oocytes. H) Co-immunoprecipitation of ATF5 in measuring POLG in mature oocytes. ATF5 and POLG were immunoprecipitated followed with SDS-PAGE separation and measured by immunoblotting. IgG was used as a negative control in immunoprecipitation (n = 5). I) Number of mutation types in D-loop of mtDNA from POLG-immunoprecipitants (n = 5). Data are presented as mean ± s.e.m. **P* < 0.05, ***P* < 0.01, ****P* < 0.001, and *****P* < 0.0001; unpaired two-tail Student's t test was used in analyses.

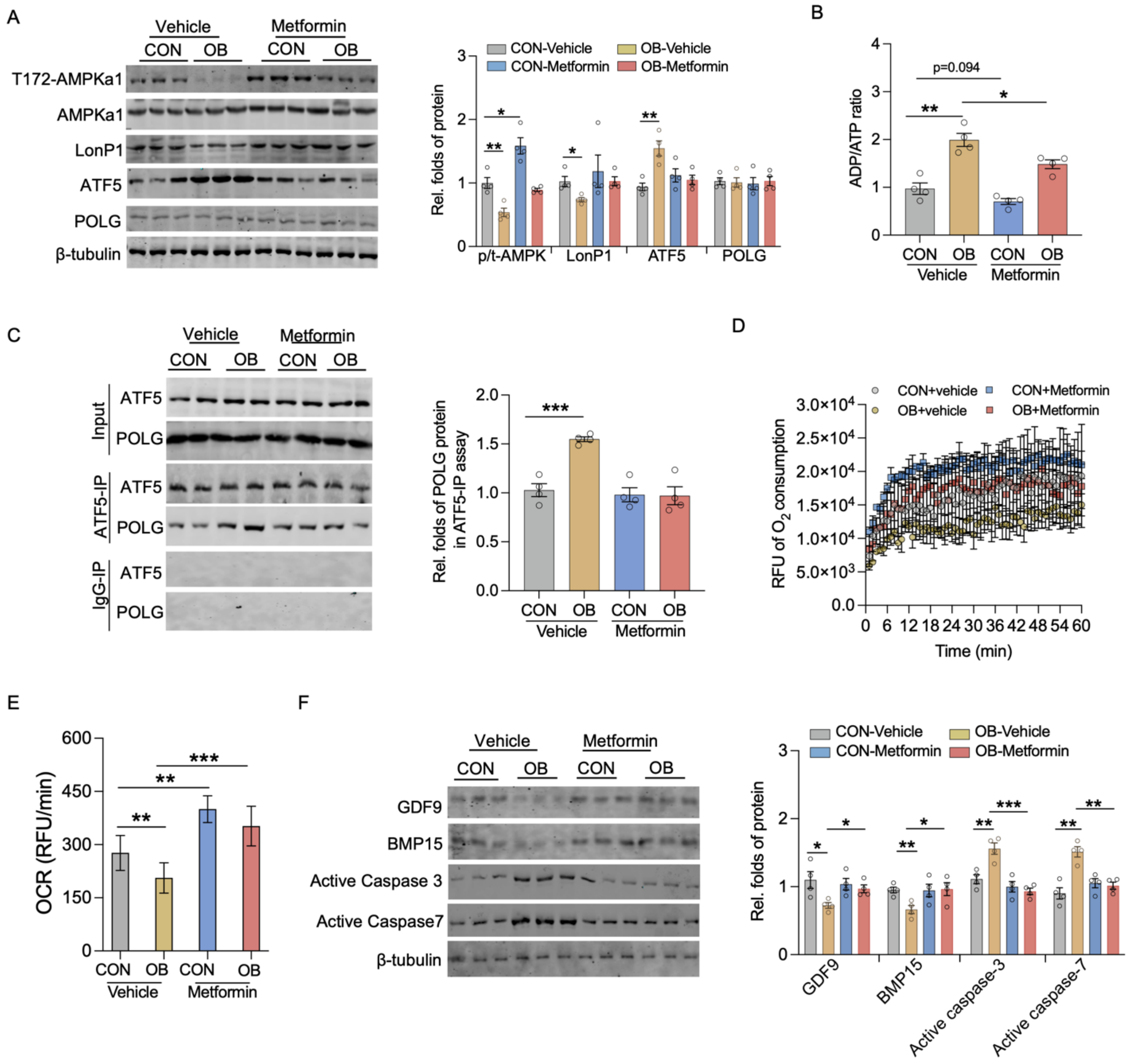

Figure S6. AMPK activation improves oocyte maturation and limiting ATF5-POLG binding in obese females. A) Oocytes were isolated in control (CON) and obese females (OB), and treated AMPKa agonists metformin *in vitro*. Immunoblotting of AMPKa, AMPKa phosphorylation at Thr-172, LonP1, ATF5 and POLG proteins in mature oocytes. GAPDH was used as loading controls (n = 4). B) ADP/ATP ratio in mature oocytes. C) Co-immunoprecipitation of ATF5 in measuring POLG in mature oocytes. ATF5 and POLG were immunoprecipitated followed with SDS-PAGE separation and measured in immunoblotting. IgG was used as a negative control in immunoprecipitation (n = 4). D, E) Oxygen

consumption was measured in mature oocytes supplemented with metformin. F) Immunoblotting of GDF9, BMP15, active-caspase3 and active-caspase7 proteins in mature oocytes. The β-tubulin was used as a loading control (n = 4). Data are presented as mean ± s.e.m. **P* < 0.05, ***P* < 0.01, ****P* < 0.001, and *****P* < 0.0001; Two-way ANOVA with Bonferroni post hoc was used in data analysis.

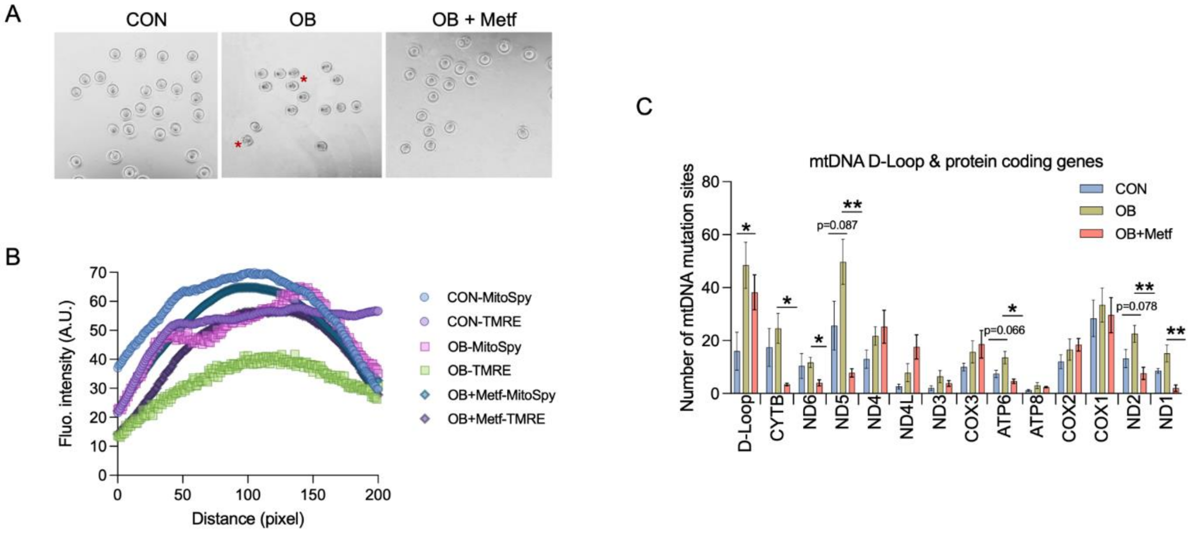

Figure S7. AMPK activation improves oocyte quality and reduces mtDNA heteroplasmy in obese females. A) The mature oocytes were collected in the fallopian tubes in control (CON), obese (OB) and obese females fed metformin (OB + Metf). Stars indicate the abnormal oocytes. B) Fluorescent intensity of MitoSpy and TMRE in mature oocytes. Intensity was quantified by Image-J. C) The number of mtDNA mutations in D-loop and protein coding genes in mature oocytes collected in CON, OB and OB+Metf females. Data are presented as mean ± s.e.m. **P* < 0.05, ***P* < 0.01, ****P* < 0.001, and *****P* < 0.0001; one-way ANOVA was used in data analysis.

Fig. 1E

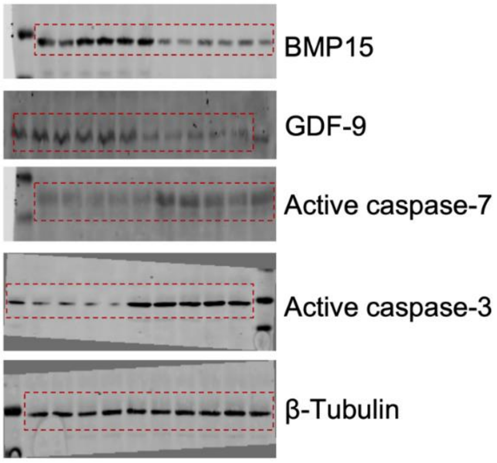

Fig. 1F

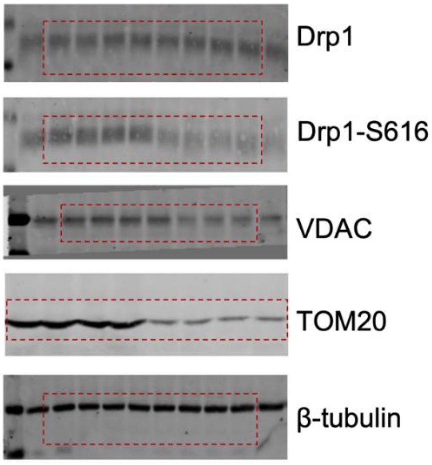

Fig. 3A

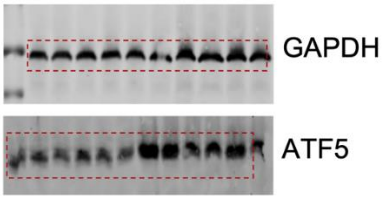

Fig. 3B

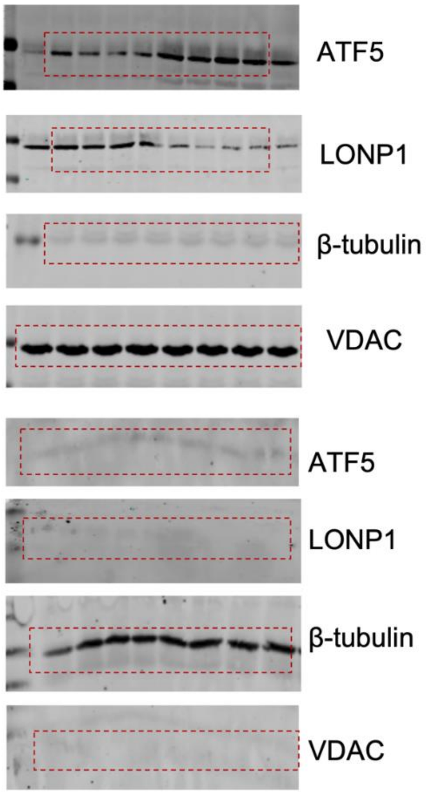

Fig. 3C

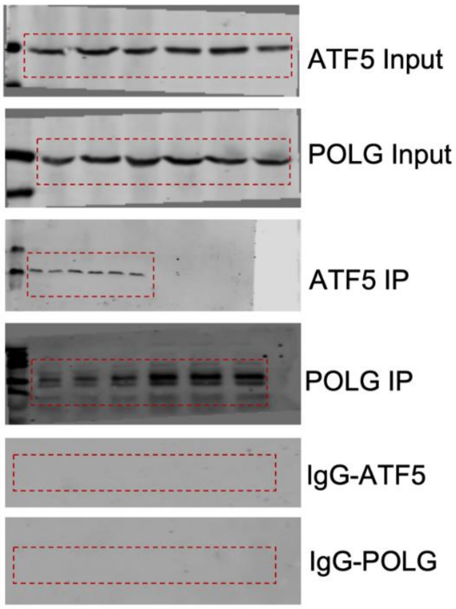

Fig. 4A

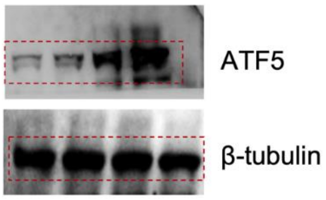

Fig. 5A

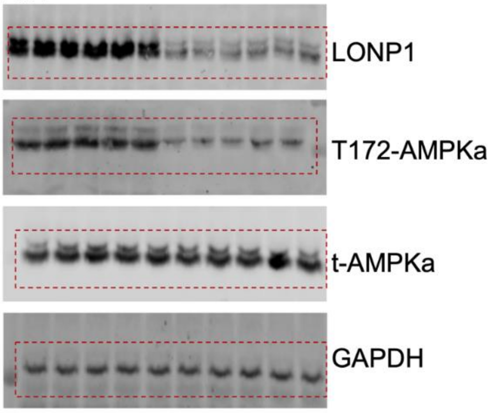

Fig. 5H

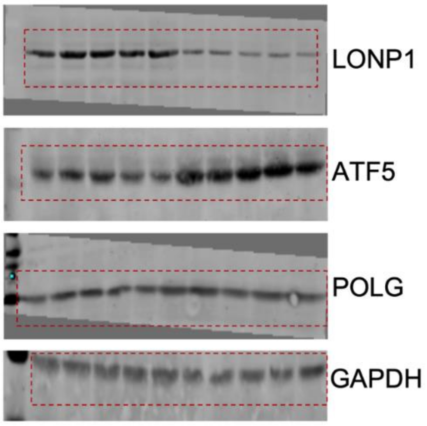

Fig. 6F

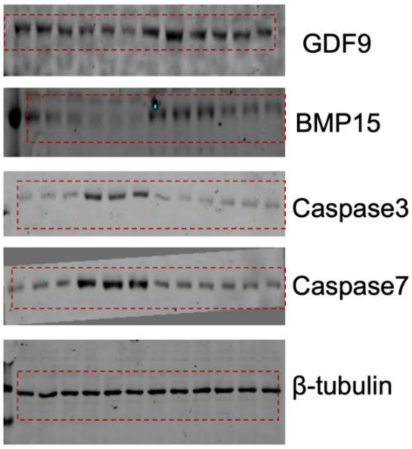

Fig. 5I

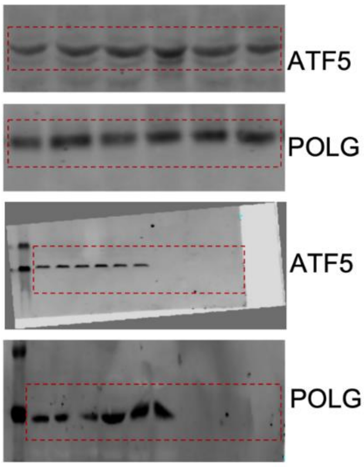

Fig. 6A

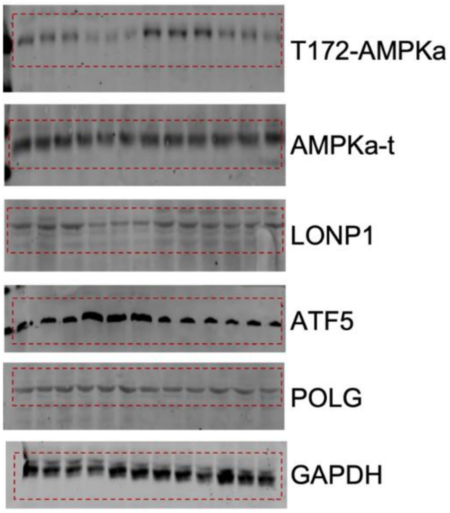

Fig. 6C

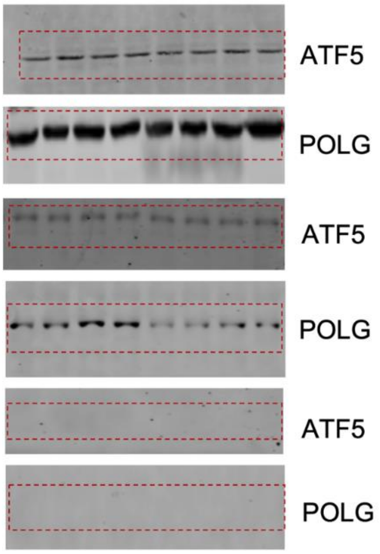

Fig. 7A

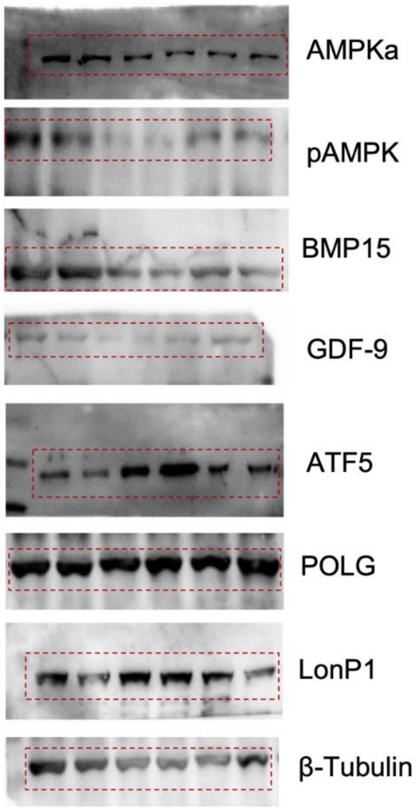

Fig. SI1C

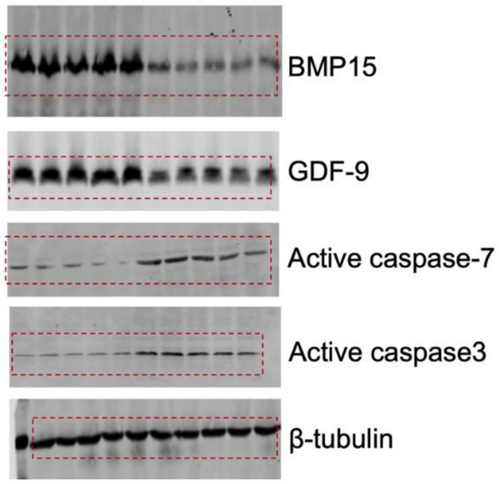

Fig. SI3B

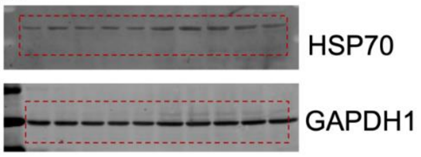

Fig. 7E

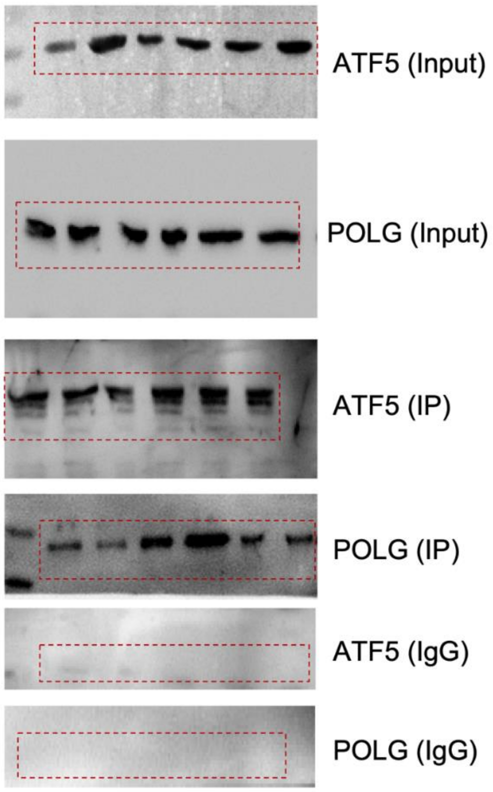

Fig. SI3D

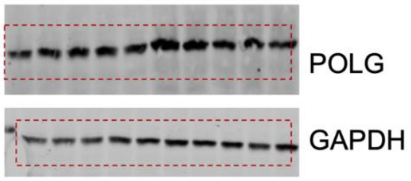

Fig. SI4B

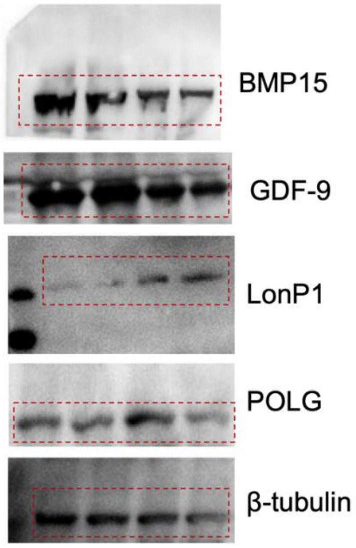

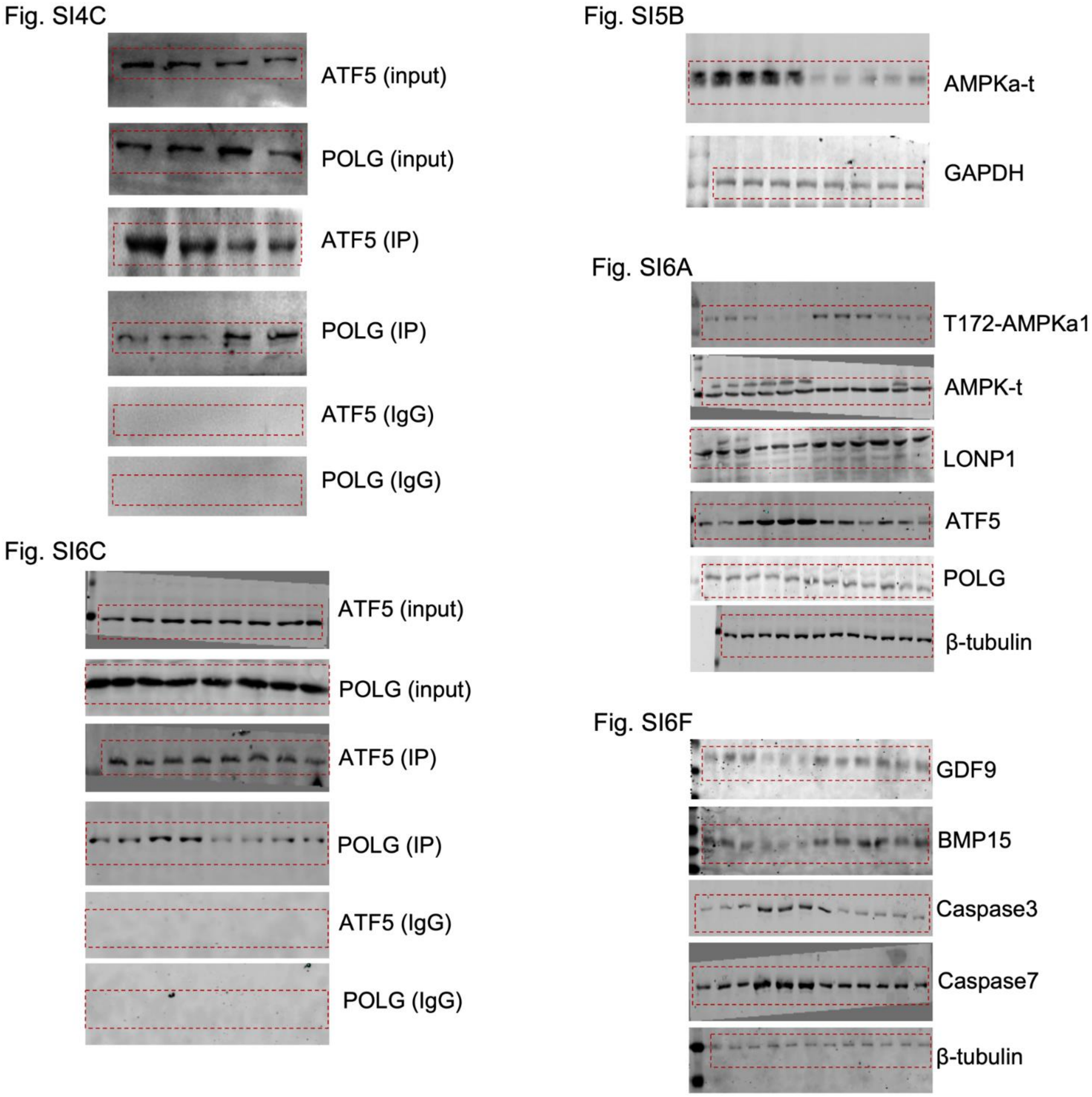

Figure S8. Uncropped immunoblotting figures shown in the study.

Table S1. List of primer sequences for gene expression analyses

Name	forward primers	reverse primers
Nd2	ACAACCCATCCCTCACTC	ATTTTGGTAAGAATCCTGTT
Nd3	CCTAACGCTAATTCTAGTTG	GACGTGCAGAGCTTGTAG
Nd4	TCCTCAGTTAGCCACATAGCA	AGGCAGAATAGGAGTGATGAT G
Nd5	ATAACCGCATCGGAGACA	TGGTAGTCATGGGTGGAG
Cytb	CTGTTCGCAGTCATAGCC	AAGAATCGGGTCAAGGTG
Cox1	ATGTTCTATCAATGGGAGC	TCTGAGTAGCGTCGTGGT
Cox3	CGAAACCACATAAATCAAG	AGTAGGCAAACAATAAGGA
ATP6	AACCTGGTGAACTACGAC	GATGTTACTGTTGCTTGAT
Clpp	GCCATTCACTGCCCAATTCC	CCTCTCCATTGCTGACTCGAT
Dnaja3	ATCAATGTGACGATCCCTGC	ATCCATGGTTCTGCCACCAG
Hspd1	CGCCCCGCAGAAATGCTT	ACTTTGCAACAGTGACCCCA
Chop	CTGAGGAGAGAGTGTTCCAG	CTAGGGACGCAGGGTCAAGA
Hspe1	CAGGAGGGAAAGGAAAGAGTG G	AGTGGAATGGCAGCTTCACG
Gtl2	TCCCCCTCCTCCACCCACCT	AAGAGCCCCTACCTGGCGCA
18s	GTAACCCGTTGAACCCCATT	CCATCCAATCGGTAGTAGCG
